# A machine learning-based model for predicting survival in patients with Rectosigmoid Cancer

**DOI:** 10.1371/journal.pone.0319248

**Published:** 2025-03-25

**Authors:** Yifei Wang, Bingbing Chen, Jinhai Yu

**Affiliations:** Department of Gastric and Colorectal Surgery, General Surgery Center, The First Hospital of Jilin University, Changchun, China; Acibadem Maslak Hospital: Acibadem Maslak Hastanesi, TÜRKIYE

## Abstract

**Background:**

The unique anatomical characteristics and blood supply of the rectosigmoid junction confer particular significance to its physiological functions and clinical surgeries. However, research on the prognosis of rectosigmoid junction cancer (RSC) is scarce, and reliable clinical prediction models are lacking.

**Methods:**

This retrospective study included 524 patients diagnosed with RSC who were admitted to the Department of Gastrointestinal and Colorectal Surgery at the First Hospital of Jilin University between January 1, 2017, and June 1, 2019. Univariate and multivariate Cox regression analyses were conducted in this study to identify independent risk factors impacting the survival of RSC patients. Subsequently, models were constructed using six different machine learning algorithms. Finally, the discrimination, calibration, and clinical applicability of each model were evaluated to determine the optimal model.

**Results:**

Through univariate and multivariate Cox regression analyses, we identified seven independent risk factors associated with the survival of RSC patients: age (HR = 1.9, 95% CI: 1.3-2.8, P = 0.001), gender (HR = 0.6, 95% CI: 0.4-0.9, P = 0.013), diabetes (HR = 2.0, 95% CI: 1.3-3.1, P = 0.002), tumor differentiation (HR = 2.1, 95% CI: 1.4-3.1, P < 0.001), tumor N stage (HR = 2.02, 95% CI: 1.2-3.4, P = 0.009), distant metastasis (HR = 4.2, 95% CI: 2.7-6.7, P < 0.001), and anastomotic leakage (HR = 2.4, 95% CI: 1.1-5.3, P = 0.034). After evaluating each model, the prediction model based on XGBoost was determined to be the optimal model, with AUC of 0.7856, 0.8484, and 0.796 at 1, 3, and 5 years. It also had the lowest Brier scores at all time points, and decision curve analysis (DCA) demonstrated the best clinical decision benefits compared to other models.

**Conclusion:**

We developed a prediction model based on the optimal machine learning, XGBoost, which can assist clinical decision-making and potentially extend the survival of patients with rectosigmoid junction cancer.

## Introduction

As a disease prevalent worldwide, colorectal cancer is the second most diagnosed cancer in women and the third in men [1]. Despite advances and the widespread adoption of screening technologies, colorectal cancer continues to be a major public health issue [2,3]. Recent statistics indicate an increase in colorectal cancer incidence among individuals younger than 55 years. During the late 1990s, colorectal cancer was the fourth leading cause of cancer deaths among individuals under 50, but it has recently emerged as the top cause of cancer mortality in men and the second highest in women [4].

Among the various tumor locations within the colorectal region, tumors located at the rectosigmoid junction hold a unique position, encompassing parts of both the rectum and the colon. Anatomically, the rectosigmoid junction is situated at the distal end of the sigmoid colon and the upper segment of the rectum. At this junction, the diameter of the intestinal lumen gradually narrows, aiding in intestinal peristalsis and fecal excretion, thus playing a crucial role in bowel function [5,6]. In terms of blood supply, the rectosigmoid junction stands in stark contrast to the sigmoid colon and rectum due to its unique vascular network. The blood supply to the rectosigmoid junction relies on the convergence of terminal branches of the inferior mesenteric artery and the superior rectal artery, forming a complex arterial anastomosis [7]. In contrast, arteries that solely supply the rectum or sigmoid colon rarely form such a dense vascular network. This distinctive anatomical feature and blood supply make the rectosigmoid junction particularly important in both physiological function and clinical surgery, setting it apart from the rectum or sigmoid colon alone.

From 1999 to 2020, the age-adjusted mortality rate (AAMR) for patients with rectosigmoid junction cancer (RSC) in the United States significantly increased [8]. Previous studies have shown that colorectal cancer exhibits considerable inter-patient heterogeneity at both histopathological and molecular levels, leading to varying disease outcomes even for patients with identical tumor stages [9]. Therefore, developing clinical prediction models capable of predicting individual prognoses for colorectal cancer is both important and meaningful. While several studies have attempted to establish clinical prediction models or other methods to predict the prognosis or subsequent treatment outcomes for colorectal cancer, there is a paucity of research focused on the prognosis of tumors at the rectosigmoid junction, and well-reported clinical prediction models are lacking [10–12].

This study aims to explore the clinical factors influencing the survival of patients with RSC and to develop an accurate survival prediction model using machine learning algorithms. By integrating clinical, pathological, and demographic data, machine learning overcomes the limitations of traditional statistical methods and offers greater precision in survival analysis. The proposed model is designed to provide data-driven support for clinicians, optimize treatment decisions, and facilitate personalized therapeutic strategies. Ultimately, it seeks to improve survival outcomes for RSC patients, highlighting the potential of machine learning in advancing cancer care.

## Patients and methods

**Fig 1:**
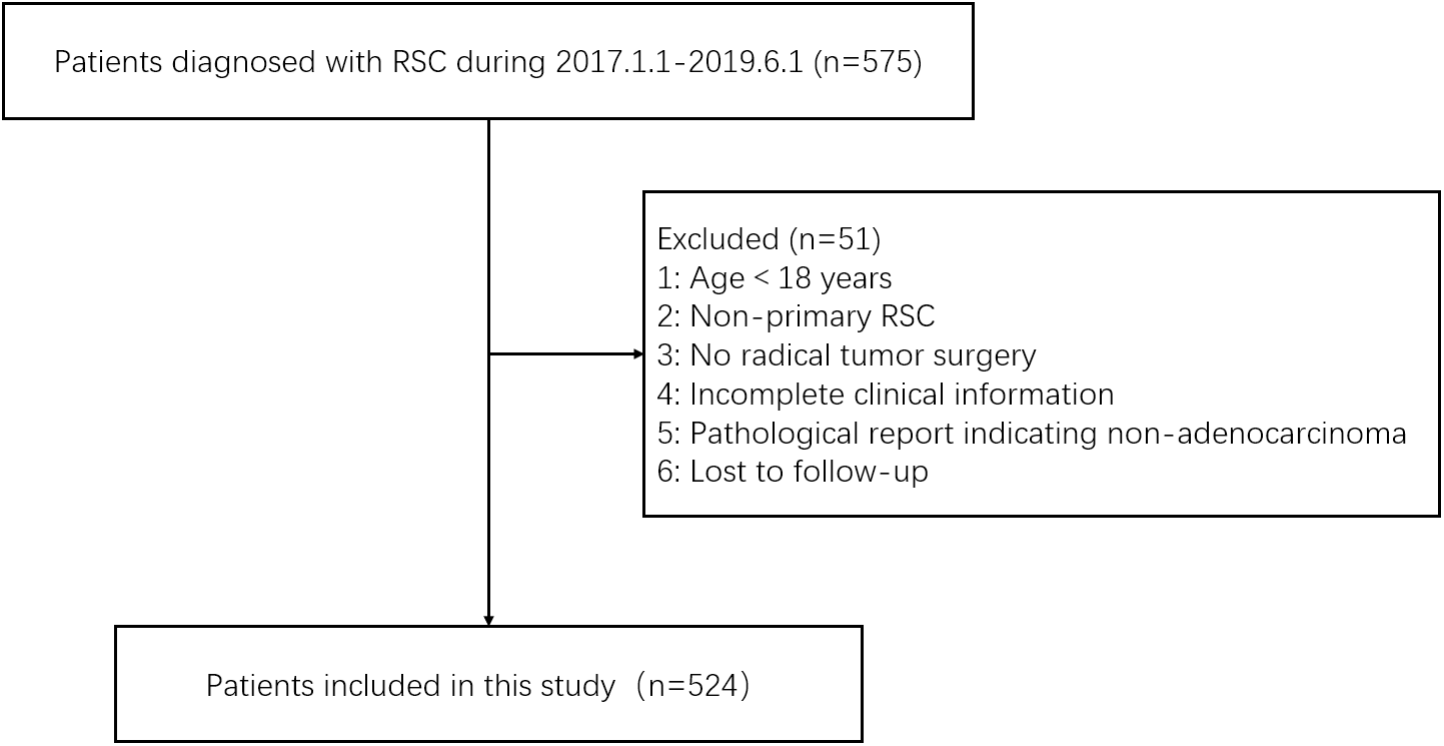
Patient screening information for this study.

This retrospective study collected and followed up on 575 patients diagnosed with RSC who were admitted to the Department of Gastrointestinal and Colorectal Surgery at the First Hospital of Jilin University between January 1, 2017, and June 1, 2019. For the definition of the rectosigmoid junction, we selected the distance of 15 cm from the anal verge using a rigid endoscope [13–16]. The inclusion criteria for our cases were as follows: (1) admission between January 1, 2017, and June 1, 2019; (2) age ≥ 18 years; (3) preoperative rigid colonoscopy with a corresponding report; (4) colonoscopy indicating the lower edge of the tumor was 15-18 cm from the anal verge; or, despite the colonoscopy indicating the lower edge of the tumor was not 15-18 cm from the anal verge, the tumor volume covered this area; or colonoscopy clearly indicating the tumor was located at the rectosigmoid junction; (5) primary RSC; (6) undergoing radical tumor resection during hospitalization; (7) surgical pathology reporting adenocarcinoma; (8) patients having complete surgical pathology information, including tumor volume, tumor stage, pericolic lymph node metastasis, perineural invasion and vascular invasion. Patients who did not meet these criteria or were missing any inclusion variables were excluded from the analysis. Additionally, we excluded 51 patients due to loss of follow-up. Based on strict adherence to the inclusion and exclusion criteria, a total of 524 patients were ultimately included in this study. Data access occurred from July 1, 2024, to July 31, 2024, with data sourced from the medical records management system of the First Hospital of Jilin University. Data retrieval was conducted after obtaining the corresponding ethics approval. The data does not contain personally identifiable information. The study was approved by the Ethics Committee of the First Hospital of Jilin University, which granted a waiver for informed consent, as this retrospective research does not involve patient privacy or any potential health risks. The study adhered to the Helsinki Declaration. Detailed patient screening information is shown in Fig 1.

We collected clinical information for patients diagnosed with RSC who were admitted to the First Hospital of Jilin University between January 1, 2017, and June 1, 2019, using the medical record collection system. The data were subsequently processed accordingly. The pathological staging used in this study followed the 8th edition of the AJCC Cancer Staging Manual. Categorical variables were encoded in S1 Table.

## Model construction and evaluation

In this study, we used six machine learning algorithms, including Extreme Gradient Boosting (XGBoost), Recursive Partitioning and Regression Trees (Rpart), Gradient Boosting Machine (GBM), Support Vector Machine (SVM), Random Survival Forest (RSF), and Lasso-Cox Model (Lasso-cox). XGBoost is a gradient boosting framework that leverages tree-based learning algorithms, has demonstrated efficacy in constructing prognostic and diagnostic medical models in recent years. Rpart is an intuitive decision tree algorithm that builds predictive models by recursively partitioning the dataset, excelling in handling both classification and regression problems. GBM is an ensemble learning method that improves model predictive performance by sequentially building multiple weak learners, with each new learner attempting to correct the errors of the previous model, thereby gradually enhancing the overall model accuracy. SVM is a widely implemented supervised learning algorithm, extensively utilized for both classification and regression tasks. It constructs a maximum margin hyperplane in high-dimensional space to effectively separate data points, possessing strong capabilities in handling nonlinear and high-dimensional data. RSF is an extension of the random forest for survival analysis. It reduces training variance and enhances ensemble and generalization by constructing multiple survival decision trees, offering high predictive accuracy and stability. Lasso-cox is a model that combines dimensionality reduction with the traditional Cox model, effectively fitting data related to survival analysis. The data were randomly split into training cohort and testing cohort with an 8:2 ratio. After identifying independent risk factors related to the survival of RSC patients through univariate and multivariate Cox regression analyses, we trained the training set data using six different machine learning algorithms. To determine the best model among these six, we conducted a comprehensive evaluation using multiple performance metrics. Subsequently, we applied these trained models to the internal validation set to assess their generalization ability and extrapolation performance. By comparing the performance of each model, we identified the final model. The evaluation of the models covered three dimensions: discrimination, calibration, and clinical applicability. Discrimination is typically assessed using the area under the receiver operating characteristic curve (AUC). The AUC values reflect the predictive ability of the model: an AUC <  0.50 indicates no predictive ability; 0.50 < AUC ≤  0.70 indicates moderate accuracy; 0.70 < AUC ≤  0.90 indicates moderate to high accuracy; and AUC >  0.90 indicates high accuracy. To evaluate the predictive accuracy of the models, we introduced the Brier score. The Brier score quantifies the mean squared error between predicted probabilities and actual outcomes; lower scores indicate better predictive performance. The unique advantage of the Brier score is that it not only assesses the accuracy of the model but also evaluates whether the predicted probabilities are consistent with the actual outcomes. For the clinical applicability of the models, we used decision curve analysis. The DCA curve integrates the preferences of patients and decision-makers into the analysis, offering a better assessment of the clinical utility of predictive models compared to the ROC curve.

### Survival analysis

Survival analysis in this study focused on overall survival (OS). The Kaplan-Meier method was used to estimate OS, and differences between groups were compared using the log-rank test. Cox proportional hazards regression was employed to identify factors independently associated with OS, with results reported as hazard ratio (HRs) and 95% confidence intervals (CIs). Variables with P-values < 0.05 in univariate Cox regression analysis were included in a multivariate Cox model to adjust for potential confounders.

### Statistical analysis

All analyses in this study were performed using R software (version 4.2.3). We have made the code used in this study available in S1 Data. The Shapiro-Wilk test was used to assess the normality of continuous variables. Continuous variables with a normal distribution were presented as mean ±  standard deviation (mean ±  SD) and compared using the independent samples t-test. Non-normally distributed continuous variables were reported as median and interquartile range (IQR) and analyzed using the Kruskal-Wallis test. Categorical variables were shown as counts (percentages) and compared using the chi-square test or Fisher’s exact test. Fisher’s exact test was used when T < 1; the corrected chi-square test was used when 1 ≤ T ≤ 5; and Pearson’s chi-square test was used when T ≥ 5. A two-sided P-value < 0.05 was considered statistically significant.

## Results

### Clinical characteristics of patients

A total of 524 patients were included in this study, randomly divided into a training cohort (n = 415) and an internal testing cohort (n = 109) (**Table 1**). The majority of RSC patients were elderly males over 60 years old (57.3%) (69.5%). A small portion of the patients had hypertension (21.2%) and/or diabetes (11.5%). Most patients underwent laparoscopic surgery during hospitalization (97.7%), while a few underwent open surgery (2.3%). The tumors were predominantly moderately to poorly differentiated (84.9%), with a small portion being well-differentiated (15.1%). The maximum diameter of most tumors was ≤ 5 cm (72.9%). Among the patients, 17.2% were in T stage I-II, while 82.8% were in T stage III-IV. 48.1% of the patients were in N stage 0, while 51.9% were in N stage 1-2. 31.1% of the patients had perineural invasion, and 48.0% had vascular invasion. The vast majority of patients had no distant metastasis at diagnosis (80.8%). Very few patients experienced anastomotic leakage (1.9%) or underwent reoperation (1.1%) postoperatively. Most patients had no history of surgery before hospitalization (73.7%). A small number of patients underwent protective stoma surgery (5.3%), while the vast majority did not (94.7%). Only a few RSC patients received radiotherapy postoperatively (4.6%), while more patients opted for chemotherapy (57.1%). The mean survival was 1664.60 days, with a standard deviation of 624.92 days.

**Table 1:** Clinical Characteristics of Patients.

	All patients (n = 524)	Alive (n = 377)	Dead (n = 147)	*P*
Age				<0.001
≤ 60	224(42.7)	186(49.3)	38(25.9)	
> 60	300(57.3)	191(50.7)	109(74.1)	
Gender				0.005
Male	364(69.5)	248(65.8)	116(78.9)	
Female	160(30.5)	129(34.2)	31(21.1)	
Hypertension				0.080
No	413(78.8)	305(80.9)	108(73.5)	
Yes	111(21.2)	72(19.1)	39(26.5)	
Diabetes				0.008
No	464(88.5)	343(91.0)	121(82.3)	
Yes	60(11.5)	34(9.0)	26(17.7)	
Surgical technique				0.042
Open	12(2.3)	5(1.3)	7(4.8)	
Laparoscopy	512(97.7)	372(98.7)	140(95.2)	
Differentiated				0.002
Moderate-Poor	445(84.9)	332(88.1)	113(76.9)	
Well	79(15.1)	45(11.9)	34(23.1)	
Tumor size				0.058
≤ 5 cm	382(72.9)	284(75.3)	98(66.7)	
> 5 cm	142(27.1)	93(24.7)	49(33.3)	
T stage				<0.001
I-II	77(14.7)	69(18.3)	8(5.4)	
III-IV	447(85.3)	308(81.7)	139(94.6)	
N stage				<0.001
0	252(48.1)	209(55.4)	43(29.3)	
1-2	272(51.9)	168(44.6)	104(70.7)	
Nerve invasion				<0.001
No	325(62.0)	259(68.7)	66(44.9)	
Yes	199(38.0)	118(31.3)	81(55.1)	
Vascular Invasion				<0.001
No	269(51.3)	215(57.0)	54(36.7)	
Yes	255(48.7)	162(43.0)	93(63.3)	
Metastasis				<0.001
No	482(92.0)	373(98.9)	109(74.1)	
Yes	42(8.0)	4(1.1)	38(25.9)	
Anastomotic leak				0.009
No	514(98.1)	374(99.2)	140(95.2)	
Yes	10(1.9)	3(0.8)	7(4.8)	
Protective stoma				0.068
No	489(93.3)	357(94.7)	132(89.8)	
Yes	35(6.7)	20(5.3)	15(10.2)	
Reoperation				1.000
No	518(98.9)	373(98.9)	145(98.6)	
Yes	6(1.1)	4(1.1)	2(1.4)	
Surgical history				0.403
No	386(73.7)	282(74.8)	104(70.7)	
Yes	138(26.3)	95(25.2)	43(29.3)	
Chemotherapy				0.270
No	225(42.9)	168(44.6)	57(38.8)	
Yes	299(57.1)	209(55.4)	90(61.2)	
Radiotherapy				0.566
No	500(95.4)	358(95.0)	142(96.6)	
Yes	24(4.6)	19(5.0)	5(3.4)	
CA199				<0.001
≤ 37 U/mL	458(87.4)	348(92.3)	110(74.8)	
> 37 U/mL	66(12.6)	29(7.7)	37(25.2)	
AFP				1.000
≤ 25 μg/L	522(99.6)	376(99.7)	146(99.3)	
> 25 μg/L	2(0.4)	1(0.3)	1(0.7)	
CEA				<0.001
≤ 5 μg/L	317(60.5)	250(66.3)	67(45.6)	
> 5 μg/L	207(39.5)	127(33.7)	80(54.4)	
Number of positive lymph nodes(mean(SD))	1.79 ± 3.76	1.16 ± 2.12	3.39 ± 5.95	<0.001
Survival(mean(SD))	1664.60(624.92)	1864.14(408.63)	1152.86(776.15)	<0.001

Abbreviations: CEA, Carcinoembryonic Antigen; SD, standard deviation; AFP, alphafetoprotein.

**Fig 2:**
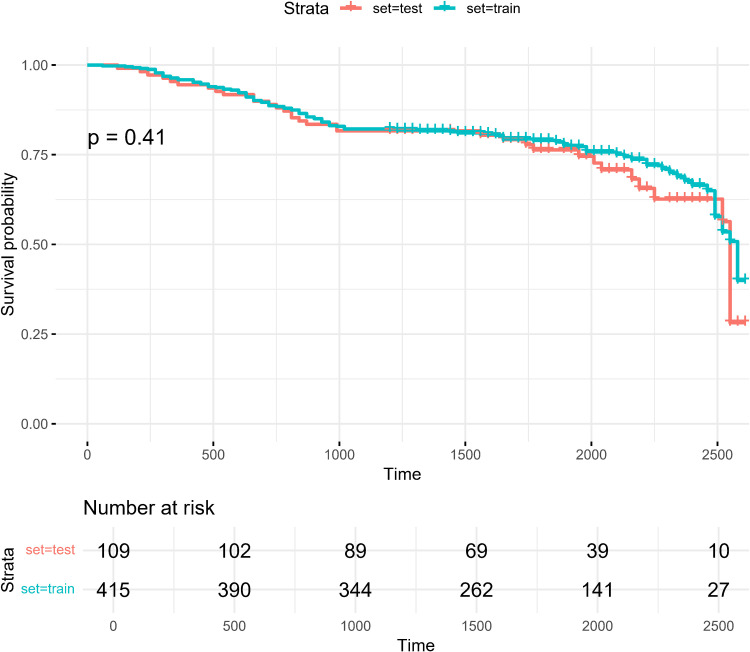
Kaplan-Meier survival curve analysis of patients in the training and testing cohorts.

The included patients were randomly divided into training and validation sets in an 8:2 ratio (**Table 2**). To verify the balance between the training and test sets, we performed Kaplan-Meier survival curve analysis on the survival probabilities of the two groups of patients (Fig 2). The results showed no significant difference in survival curves between the test set and the training set (*P* = 0.41), indicating that the survival outcomes of patients in the training and test sets were statistically similar. This similarity suggests that our dataset is balanced, ensuring that the model to be constructed will have consistent predictive performance across both datasets. Through this analysis, we can ensure that the training and testing data are comparable.

**Table 2:** Clinical and pathological characteristics of the train set and test set.

	Train			Test		
	All patients (n = 415)	Alive (n = 303)	Dead (n = 112)	All patients (n = 109)	Alive (n = 74)	Dead (n = 35)
Age						
≤ 60	174(41.9)	147(48.5)	27(24.1)	50(45.9)	39(52.7)	11(31.4)
> 60	241(58.1)	156(51.5)	85(75.9)	59(54.1)	35(47.3)	24(68.6)
Gender						
Male	286(68.9)	200(66.0)	86(76.8)	78(71.6)	48(64.9)	30(85.7)
Female	129(31.1)	103(34.0)	26(23.2)	31(28.4)	26(35.1)	5(14.3)
Hypertension						
No	320(77.1)	241(79.5)	79(70.5)	93(85.3)	64(86.5)	29(82.9)
Yes	95(22.9)	62(20.5)	33(29.5)	16(14.7)	10(13.5)	6(17.1)
Diabetes						
No	365(88.0)	272(89.8)	93(83.0)	99(90.8)	71(95.9)	28(80.0)
Yes	50(12.0)	31(10.2)	19(17.0)	10(9.2)	3(4.1)	7(20.0)
Surgical technique						
Open	7(1.7)	2(0.7)	5(4.5)	5(4.6)	3(4.1)	2(5.7)
Laparoscopy	408(98.3)	301(99.3)	107(95.5)	104(95.4)	71(95.9)	33(94.3)
Differentiated						
Moderate-Poor	349(84.1)	265(87.5)	84(75.0)	96(88.1)	67(90.5)	29(82.9)
Well	66(15.9)	38(12.5)	28(25.0)	13(11.9)	7(9.5)	6(17.1)
Tumor size						
≤ 5 cm	300(72.3)	228(75.2)	72(64.3)	82(75.2)	56(75.7)	26(74.3)
> 5 cm	115(27.7)	75(24.8)	40(35.7)	27(24.8)	18(24.3)	9(25.7)
T stage						
I-II	65(15.7)	58(19.1)	7(6.2)	12(11.0)	11(14.9)	1(2.9)
III-IV	350(84.3)	245(80.9)	105(93.8)	97(89.0)	63(85.1)	34(97.1)
N stage						
0	194(46.7)	160(52.8)	34(30.4)	58(53.2)	49(66.2)	9(25.7)
1-2	221(53.3)	143(47.2)	78(69.6)	51(46.8)	25(33.8)	26(74.3)
Nerve invasion						
No	261(62.9)	208(68.6)	53(47.3)	64(58.7)	51(68.9)	13(37.1)
Yes	154(37.1)	95(31.4)	59(52.7)	45(41.3)	23(31.1)	22(62.9)
Vascular Invasion						
No	212(51.1)	167(55.1)	45(40.0)	57(52.3)	48(64.9)	9(25.7)
Yes	203(48.9)	136(44.9)	67(59.8)	52(47.7)	26(35.1)	26(74.3)
Metastasis						
No	384(92.5)	299(98.7)	85(75.9)	98(89.9)	74(100.0)	24(68.6)
Yes	31(7.5)	4(1.3)	27(24.1)	11(10.1)	0(0.0)	11(31.4)
Anastomotic leak						
No	405(97.6)	300(99.0)	105(93.8)	0(0.0)	0(0.0)	0(0.0)
Yes	10(2.4)	3(1.0)	7(6.2)	109(100.0)	74(100.0)	35(100.0)
Protective stoma						
No	389(93.3)	287(94.7)	102(91.1)	100(91.7)	70(94.6)	30(85.7)
Yes	26(6.3)	16(5.3)	10(8.9)	9(8.3)	4(5.4)	5(14.3)
Reoperation						
No	409(98.6)	299(98.7)	110(98.2)	0(0.0)	0(0.0)	0(0.0)
Yes	6(1.4)	4(1.3)	2(1.8)	109(100.0)	74(100.0)	35(100.0)
Surgical history						
No	108(26.0)	76(25.1)	32(28.6)	30(27.5)	19(25.7)	11(31.4)
Yes	307(74.0)	227(74.9)	80(71.4)	79(72.5)	55(74.3)	24(68.6)
Chemotherapy						
No	181(43.6)	131(43.2)	50(44.6)	44(40.6)	37(50.0)	7(20.0)
Yes	234(56.4)	172(56.8)	62(55.4)	65(59.6)	37(50.0)	28(80.0)
Radiotherapy						
No	397(95.7)	290(95.7)	107(95.5)	103(94.5)	68(91.9)	35(100.0)
Yes	18(4.3)	13(4.3)	5(4.5)	6(5.5)	6(8.1)	0(0.0)
CA199						
≤ 37 U/mL	361(87.0)	277(91.4)	84(75.0)	97(89.0)	71(95.9)	26(74.3)
> 37 U/mL	54(13.0)	26(8.6)	28(25.0)	12(11.0)	3(4.1)	9(25.7)
AFP						
≤ 25 μg/L	414(99.8)	302(99.7)	112(100.0)	108(99.1)	74(100.0)	34(97.1)
> 25 μg/L	1(0.2)	1(0.3)	0(0.0)	1(0.9)	0(0.0)	1(2.9)
CEA						
≤ 5 μg/L	248(59.8)	196(64.7)	52(46.4)	69(63.3)	54(73.0)	15(42.9)
> 5 μg/L	167(40.2)	107(35.3)	60(53.6)	40(36.7)	20(27.0)	20(57.1)
Survival((mean(SD))	1664.31(621.42)	1865.45(408.49)	1120.18(759.69)	1665.69(640.96)	1858.78(411.95)	1257.43(829.39)

Abbreviations: CEA, Carcinoembryonic Antigen; SD, standard deviation; AFP, alphafetoprotein.

### Univariate and multivariate cox regression analysis of survival in RSC patients

We applied both univariate and multivariate Cox regression analyses to pinpoint the risk factors linked to the survival of patients with rectosigmoid junction cancer (RSC) in the training set (**Table 3**). The results of the univariate Cox regression showed that age, gender, diabetes, tumor differentiation grade, tumor T stage, tumor N stage, perineural invasion, vascular invasion, distant metastasis, anastomotic leakage, protective stoma, CA199 level, and CEA level were all associated with cancer survival in RSC patients (*P* < 0.05). After multivariate regression analysis, we identified seven independent risk factors associated with the survival of RSC patients: age (HR = 1.9, 95% CI: 1.3-2.8, P = 0.001), gender (HR = 0.6, 95% CI: 0.4-0.9, *P* = 0.013), diabetes (HR = 2.0, 95% CI: 1.3-3.1, *P* = 0.002), tumor differentiation grade (HR = 2.1, 95% CI: 1.4-3.1, P < 0.001), tumor N stage (HR = 2.02, 95% CI: 1.2-3.4, *P* = 0.009), distant metastasis (HR = 4.2, 95% CI: 2.7-6.7, *P* < 0.001), and anastomotic leakage (HR = 2.4, 95% CI: 1.1-5.3, *P* = 0.034).

**Table 3:** Univariate and multivariate COX analyses of survival in rectosigmoid cancer.

	Univariate Analysis		Multivariate Analysis	
	HR (95% CI)	*P*	HR (95% CI)	*P*
Age				
≤ 60	Ref	<0.001	Ref	0.001
> 60	2.2 (1.5-3.2)		1.9(1.3-2.8)	
Gender				
Male	Ref	0.005	Ref	0.013
Female	0.6 (0.4-0.8)		0.6(0.4-0.9)	
Hypertension				
No	Ref	0.200	Ref	
Yes	1.3 (0.9-1.8)			
Diabetes				
No	Ref	0.0071	Ref	0.002
Yes	1.8 (1.2-2.7)		2.0(1.3-3.1)	
Surgical technique				
Open	Ref	0.210		
Laparoscopy	0.6 (0.3-1.3)			
Differentiated				
Well	Ref	<0.001	Ref	<0.001
Moderate-Poor	2.2 (1.5-3.3)		2.1(1.4-3.1)	
Tumor size				
≤ 5 cm	Ref	0.057		
> 5 cm	1.4 (1.0-2.0)			
T stage				
I-II	Ref	0.003	Ref	0.360
III-IV	3.0 (1.5-6.1)		1.4(0.7-3.1)	
N stage				
0	Ref	<0.001	Ref	0.009
1-2	2.4 (1.7-3.5)		2.02(1.2-3.4)	
Nerve invasion				
No	Ref	<0.001	Ref	0.050
Yes	2.3 (1.7-3.2)		1.5 (1.0-2.2)	
Vascular Invasion				
No	Ref	<0.001	Ref	0.110
Yes	1.9 (1.4-2.7)		0.7(0.4-1.1)	
Metastasis				
No	Ref	<0.001	Ref	<0.001
Yes	7 (4.8-10.0)		4.2(2.7-6.7)	
Anastomotic leak				
No	Ref	0.008	Ref	0.034
Yes	2.8 (1.3-6.0)		2.4(1.1-5.3)	
Protective stoma				
No	Ref	0.049	Ref	0.566
Yes	1.7 (1.0-2.9)		1.12(0.7-2.1)	
Reoperation				
No	Ref	0.690		
Yes	1.3 (0.3-5.4)			
Surgical history				
No	Ref	0.920		
Yes	0.98 (0.7-1.4)			
Chemotherapy				
No	Ref	0.420		
Yes	1.1 (0.8-1.6)			
Radiotherapy				
No	Ref	0.510		
Yes	0.7 (0.3-1.8)			
CA199				
≤ 37 U/mL	Ref	<0.001	Ref	0.452
> 37 U/mL	2.9 (2.0-4.2)		1.2(0.8-1.9)	
AFP				
≤ 25 μg/L	Ref	0.350		
> 25 μg/L	2.6 (0.4-18)			
CEA				
≤ 5 μg/L	Ref	<0.001	Ref	0.495
> 5 μg/L	1.9 (1.4-2.7)		1.1(0.8-1.7)	

Abbreviations: CEA, Carcinoembryonic Antigen; SD, standard deviation; AFP, alphafetoprotein; HR, Hazard Ratio; CI, Confidence Interval.

### Evaluation of the predictive performance of six machine learning models for RSC patient survival

**Fig 3:**
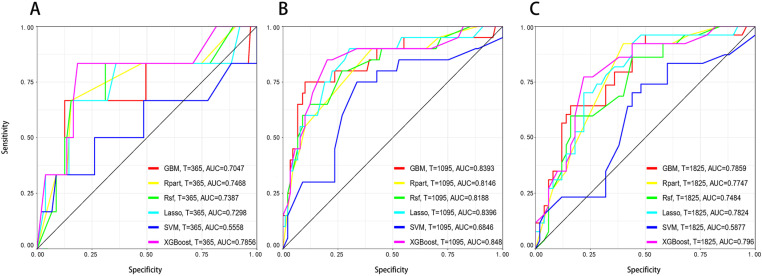
AUC of six machine learning models at 1 year, 3 years, and 5 years. Abbreviations: AUC, Area under the Curve; XGBoost, Extreme Gradient Boosting; Rpart,Recursive Partitioning and Regression Trees; GBM, Gradient Boosting Machine; SVM, Support Vector Machine; RSF, Random Survival Forest; Lasso-Cox, Lasso-Cox Model.

We used ROC curves to evaluate the predictive performance of six machine learning algorithms for overall survival at 1 year, 3 years, and 5 years (Fig 3). For the 1-year survival prediction, XGBoost (AUC = 0.786), Rpart (AUC = 0.747), RSF (AUC = 0.739), Lasso-Cox (AUC = 0.730), GBM (AUC = 0.705), and SVM (AUC = 0.556). For the 3-year survival prediction, XGBoost (AUC = 0.848), Lasso-Cox (AUC = 0.840), GBM (AUC = 0.839), RSF (AUC = 0.819), Rpart (AUC = 0.815), and SVM (AUC = 0.685). For the 5-year survival prediction, XGBoost (AUC = 0.796), GBM (AUC = 0.786), Lasso-Cox (AUC = 0.782), Rpart (AUC = 0.775), RSF (AUC = 0.748), and SVM (AUC = 0.588). These results indicate that XGBoost model consistently exhibited the strongest predictive capability across short-term, medium-term, and long-term survival predictions. It outperformed the other models in terms of predictive accuracy, demonstrating its stability and reliability for predicting the survival of RSC patients.

**Fig 4:**
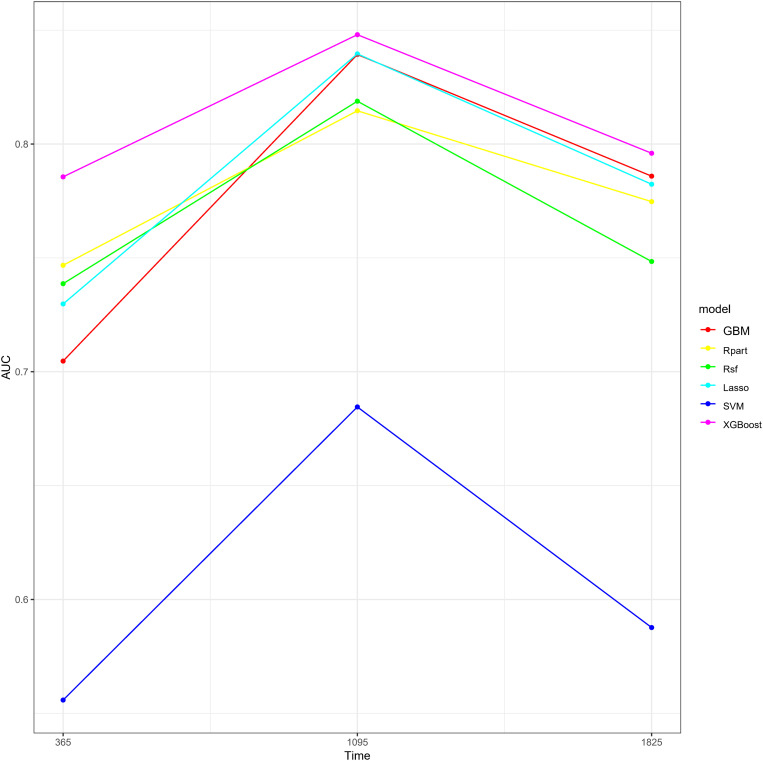
Continuous comparison of AUC of six machine learning models at different time points (1 year, 3 years, and 5 years). Abbreviations:AUC, Area under the Curve; XGBoost, Extreme Gradient Boosting; Rpart,Recursive Partitioning and Regression Trees; GBM, Gradient Boosting Machine; SVM, Support Vector Machine; RSF, Random Survival Forest; Lasso-Cox, Lasso-Cox Model.

**Fig 5:**
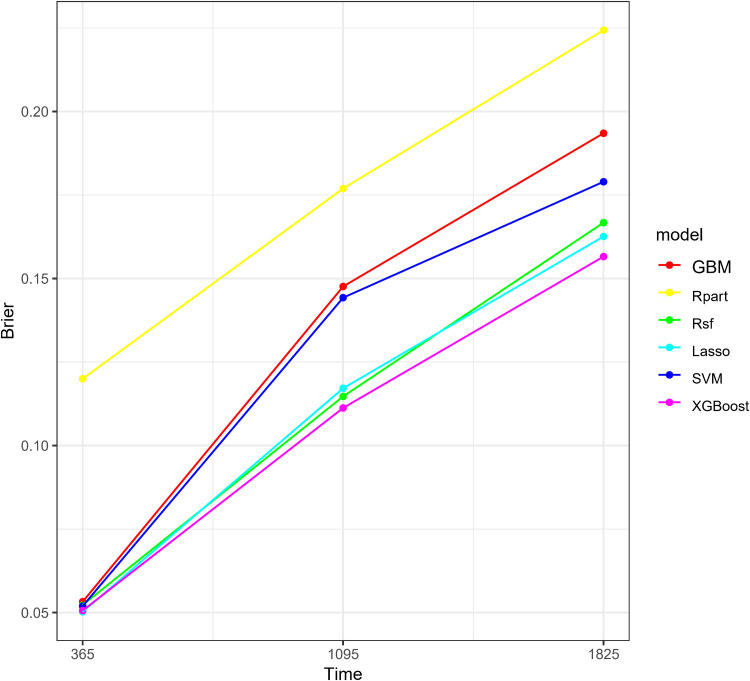
Brier score curves of six machine learning models at 1 year, 3 years, and 5 years. Abbreviations: XGBoost, Extreme Gradient Boosting; Rpart,Recursive Partitioning and Regression Trees; GBM, Gradient Boosting Machine; SVM, Support Vector Machine; RSF, Random Survival Forest; Lasso-Cox, Lasso-Cox Model.

To visually demonstrate the predictive performance of each model at different time points, we plotted the continuous AUC comparisons of the models at 1 year, 3 years, and 5 years (Fig 4). The results showed that the AUC values of the XGBoost model were higher than other models at all time points, indicating its superior performance in predicting short-term, medium-term, and long-term survival. We also displayed the Brier score curves of the six machine learning models at different time points (1 year, 3 years, and 5 years). It indicated that the Brier scores of the XGBoost model were the lowest at all time points, indicating that its predictive performance was superior to that of the other models (Fig 5).

**Fig 6:**
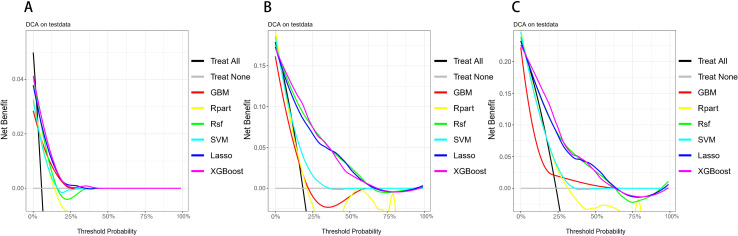
Decision curves for six machine learning models at 1 year, 3 years, and 5 years. Abbreviations: XGBoost, Extreme Gradient Boosting; Rpart,Recursive Partitioning and Regression Trees; GBM, Gradient Boosting Machine; SVM, Support Vector Machine; RSF, Random Survival Forest; Lasso-Cox, Lasso-Cox Model.

To evaluate the clinical applicability of the models, we plotted the decision curves for each model at 1 year, 3 years, and 5 years (Fig 6). DCA revealed that the XGBoost model demonstrated the highest net benefit across most thresholds for 1-year, 3-year, and 5-year survival predictions, indicating its superior practical value in clinical decision-making. Although other models also performed well at certain specific thresholds, they were generally less stable and accurate compared to the XGBoost model.

Overall, the XGBoost model demonstrated the best performance in predicting the survival of RSC patients. It significantly outperformed the other models in terms of discrimination ability (AUC values), predictive accuracy and calibration (Brier scores), and net benefit in clinical decision-making (decision curve analysis). These results indicate that the XGBoost model not only has high predictive performance but also strong clinical utility and reliability, making it the optimal choice for predicting the survival of RSC patients.

## Discussion

In this study, we analyzed independent risk factors associated with the survival of RSC patients. Subsequently, we constructed and evaluated survival prediction models based on six advanced machine learning algorithms. Our results showed that the Extreme Gradient Boosting (XGBoost) model performed the best across all evaluation metrics, demonstrating its great potential in clinical prediction models. Although several studies have previously focused on constructing prognostic models for colorectal cancer, these studies typically categorized cancers at the rectosigmoid junction as either rectal or sigmoid colon cancers, without separate investigation. In contrast, we have innovatively applied machine learning algorithms to construct a prognostic model specifically for RSC patients. Compared to traditional nomogram models, our machine learning-based predictive model demonstrates significant improvements in both performance and applicability. To our knowledge, this is the first machine learning-based prediction model specifically constructed for the survival of RSC patients.

Through univariate and multivariate regression analyses, we identified seven independent risk factors affecting the survival of RSC patients: age, gender, diabetes, tumor differentiation grade, tumor N stage, distant metastasis, and anastomotic leakage. Age plays a significant role in predicting the survival of RSC patients. Our study found that patients over 60 years old had poorer prognoses. Elderly patients often exhibit higher mortality rates and shorter survival periods when diagnosed with colorectal cancer due to decreased immune function, multiple comorbidities, and poorer tolerance to treatment [17]. Additionally, older patients tend to have a lower tolerance for treatments such as chemotherapy and radiotherapy, increasing the likelihood of complications, which can adversely affect survival [18]. Therefore, when formulating treatment plans for elderly RSC patients, it is crucial to pay closer attention to their overall health status. It may be necessary to consider reducing the invasiveness of surgery and subsequent treatments.

Among the 524 patients included in the study, female patients accounted for only 30.5% of RSC cases. From a physiological perspective, women may have a relatively lower risk of developing colorectal cancer due to the protective effects of hormones such as estrogen. Estrogen is believed to have anti-inflammatory and anti-tumor properties, inhibiting tumor occurrence and progression. Estrogen and its receptors play crucial roles in regulating autoimmune diseases and anti-tumor immunity by influencing the interactions between tumor cells, immune cells, and the tumor microenvironment, thereby modulating tumor immunity [19]. However, our study indicates that once diagnosed with RSC, the prognosis for women is not significantly better than that for men. This may be related to the biological characteristics of the tumor and the response of female patients to treatment [20]. Differences between males and females in cancer progression and treatment response need to be considered when developing personalized treatment plans. For example, adjustments in chemotherapy dosage or the selection of different drug combinations may be necessary to account for varying biological responses in male and female patients.

Diabetes is considered an important prognostic factor for RSC patients. Previous studies have shown that a hyperglycemic environment promotes tumor cell growth and spread through various mechanisms. For example, hyperglycemia can enhance tumor cell proliferation and survival by activating insulin receptors and insulin-like growth factor receptors (IGF-IR) [21]. Hyperglycemia may also accelerate tumor progression by inhibiting the activity of key immune cells, such as natural killer (NK) cells, thereby allowing tumor cells to evade immune surveillance [22]. Diabetic patients often have other metabolic disorders and chronic diseases, which can exacerbate their condition and increase the risk of complications during treatment, leading to poor prognosis and affecting survival [23]. For RSC patients with diabetes, it is crucial to closely monitor and manage their blood glucose levels throughout the cancer treatment process to minimize the adverse effects of diabetes on the effectiveness of cancer therapy.

In our study, the tumor differentiation grade significantly influenced the survival of RSC patients. It is a crucial indicator of malignancy. Highly differentiated tumor cells resemble normal cells, proliferate and spread more slowly, and generally have a better prognosis. In contrast, moderately to poorly differentiated tumor cells have higher proliferative capacity and invasiveness, leading to poorer outcomes [24]. Tumor N stage reflects the extent of lymph node involvement by the tumor, which is a critical parameter for prognosis evaluation. A higher N stage indicates a broader range of tumor invasion and worse prognosis [25]. Lymph node metastasis is an early sign of tumor spread, often indicating a higher aggressiveness and risk of dissemination [26]. Distant metastasis is one of the key factors affecting the prognosis of RSC patients. It typically signifies that the tumor has spread beyond its primary site through the blood or lymphatic systems to other organs, significantly increasing the complexity and difficulty of treatment [27]. Our study shows that patients with distant metastasis have markedly poorer prognoses compared to those without. This is likely because patients with distant metastasis usually require more comprehensive and individualized treatment strategies, involving combinations of surgery, chemotherapy, and radiotherapy. Therefore, for RSC patients with low differentiation, high N staging, and distant metastases, more aggressive postoperative treatments may be required, including high-dose chemotherapy or combination therapy, to improve survival rates.

Anastomotic leakage (AL) is a common postoperative complication that significantly impacts the prognosis of RSC patients. It substantially increases the incidence of postoperative complications, prolongs hospitalization, and is associated with higher mortality rates. Additionally, AL increases the risk of infection, further affecting patient recovery and long-term survival [28]. Preventing and promptly managing anastomotic leakage is crucial for improving patient outcomes. In the surgical treatment of RSC, meticulous anastomotic techniques and strict postoperative monitoring measures are essential.

Notably, in our multivariate analysis, the P-value for perineural invasion was 0.05, indicating its marginal statistical significance. Perineural invasion, a pathological feature, has been shown to correlate with poor prognosis in various cancers [29,30]. Its presence typically signifies higher tumor aggressiveness and greater potential for spread, leading to higher recurrence rates and poorer survival outcomes. Additionally, perineural invasion may increase the risk of local recurrence and distant metastasis, further affecting overall prognosis. Although perineural invasion was not included in our model, its potential clinical significance and supporting literature suggest that future research should explore its impact on RSC patient survival and validate its value as a prognostic indicator. This could assist in more comprehensive assessment and management of RSC treatment plans, enhancing long-term survival rates.

Finally, although our study demonstrated that the XGBoost model performed the best in predicting the survival of RSC patients, there are some limitations that need to be discussed. Due to the constraints of a retrospective study, we were unable to conduct uniform genetic sequencing examinations on the patients, resulting in the absence of genetic variables in our clinical data. Additionally, our study data were derived from a single institution, which may have regional and sample size limitations. Future research could incorporate multicenter data to further optimize the XGBoost model or combine it with other techniques to enhance its predictive performance and application value. Although our internal validation has demonstrated high accuracy, external validation remains crucial. We plan to continue refining and upgrading our predictive model in subsequent studies to ensure its broader applicability and reliability.

## Conclusion

We developed a prediction model based on the optimal machine learning algorithm, XGBoost, which can assist clinical decision-making and potentially extend the survival of patients with rectosigmoid junction cancer.

## Supporting information

S1 Table De-identified raw clinical dataset of RSC patients. (XLSX)

S1 Data The code used in this study. (DOCX)

## Abbreviations

RSC: rectosigmoid cancer
AAMR: age-adjusted mortality rate
CEA: carcinoembryonic Antigen
XGBoost: extreme gradient boosting
Rpart: recursive partitioning and regression trees
GBM: gradient boosting machine
SVM: support vector machine
RSF: random survival forest
Lasso-Cox: lasso-cox model
AUROC: area under the receiver operating characteristic curve
AUC: area under the curve
DCA: decision curve analysis
IQR: interquartile range
HR: hazard ratio
CI: confidence interval
OS: overall survival
NK: natural killer (cells)
