# Supplementary material for: A machine learning-based model for predicting survival in patients with Rectosigmoid Cancer
Source: PLoS One. 2025 Mar 25;20(3):e0319248. doi: 10.1371/journal.pone.0319248 (PMC11936176; doi:10.1371/journal.pone.0319248)
Supplement: S1 Data — (DOCX) [file pone.0319248.s002.docx]

**Univariate and multivariate Cox regression analyses**

library(rms)

library("survival")

library("tableone")

data<- readr::read_csv("data.csv")

data=data[1:23]

for(i in c(1:21)){

data[[i]] <- factor(data[[i]])

}

myVars=colnames(data)[1:22]

catVars=colnames(data)[1:21]

tab2 <- CreateTableOne(vars = myVars, data =data, strata = "status" ,factorVars = catVars,addOverall = T)

tab2=print(tab2)

write.csv(tab2," Baseline Table.csv")

tab3 <- CreateTableOne(vars = myVars, data =traindata, strata = "status" ,factorVars = catVars,addOverall = T)

tab3=print(tab3)

write.csv(tab3," Training Set Baseline Table.csv")

tab4 <- CreateTableOne(vars = myVars, data =testdata, strata = "status" ,factorVars = catVars,addOverall = T)

tab4=print(tab4)

write.csv(tab4," Test Set Baseline Table.csv")

res.cox <- coxph(Surv(rfstime, status) ~ Sex, data = data)

res.cox

## Call:

## coxph(formula = Surv(time, status) ~ age, data = pbc)

##

## coef exp(coef) se(coef) z p

## age -0.08115 0.92205 0.02305 -3.52 0.000431

##

## Likelihood ratio test=14.04 on 1 df, p=0.0001786

## n= 257, number of events= 25

summary(res.cox)

## Call:

## coxph(formula = Surv(time, status) ~ age, data = pbc)

##

## n= 257, number of events= 25

##

## coef exp(coef) se(coef) z Pr(>|z|)

## age -0.08115 0.92205 0.02305 -3.52 0.000431 ***

## ---

## Signif. codes: 0 ‘***’ 0.001 ‘**’ 0.01 ‘*’ 0.05 ‘.’ 0.1 ‘ ’ 1

##

## exp(coef) exp(-coef) lower .95 upper .95

## age 0.9221 1.085 0.8813 0.9647

##

## Concordance= 0.712 (se = 0.042 )

## Likelihood ratio test= 14.04 on 1 df, p=2e-04

## Wald test = 12.39 on 1 df, p=4e-04

## Score (logrank) test = 13.16 on 1 df, p=3e-04

# Summarize the characteristics of univariate Cox regression analysis.

covariates <-colnames(data)[1:21]

# For each variable, construct the formula for survival analysis.

univ_formulas <- sapply(covariates,

function(x) as.formula(paste('Surv(rfstime, status)~', x)))

# Perform Cox regression analysis for each feature in a loop.

univ_models <- lapply( univ_formulas, function(x){coxph(x, data = data)})

# Extract the HR (Hazard Ratio), 95% confidence interval, and p-value.

univ_results <- lapply(univ_models,

function(x){

x <- summary(x)

# Obtain the p-value.

p.value<-signif(x$wald["pvalue"], digits=2)

# Obtain the HR

HR <-signif(x$coef[2], digits=2);

# Obtain the 95% CI

HR.confint.lower <- signif(x$conf.int[,"lower .95"], 2)

HR.confint.upper <- signif(x$conf.int[,"upper .95"],2)

HR <- paste0(HR, " (",

HR.confint.lower, "-", HR.confint.upper, ")")

res<-c(p.value,HR)

names(res)<-c("p.value","HR (95% CI for HR)")

return(res)

})

# Convert to a data frame and transpose it.

res <- t(as.data.frame(univ_results, check.names = FALSE))

as.data.frame(res)

write.csv(res," Univariate COX regression.csv")

## p.value HR (95% CI for HR)

## age 0.00043 0.92 (0.88-0.96)

## sex 0.38 0.58 (0.17-1.9)

## bili 8.6e-06 1.2 (1.1-1.3)

## albumin 0.02 0.3 (0.11-0.83)

## copper 0.00014 1 (1-1)

## alk.phos 0.71 1 (1-1)

## ast 0.061 1 (1-1)

## trig 0.073 1 (1-1)

## platelet 0.05 1 (1-1)

## protime 0.42 0.8 (0.46-1.4)

## stage 0.0059 2 (1.2-3.3)

library("gtsummary")

res.cox <- coxph(Surv(rfstime, status) ~ Age +Sex+ Diabetes + Grade + T + N+

PI+MG+M+Leakage+protective.stoma+Ca199+CEA, data = data)

cox=summary(res.cox)

mul_HR <- round(cox$coefficients[, 2], 2)

mul_PValue <- round(cox$coefficients[, 5], 4)

mul_CI1 <- round(cox$conf.int[, 3], 2)

mul_CI2 <- round(cox$conf.int[, 4], 2)

mul_CI95 <- paste(mul_CI1, '-', mul_CI2)

b=mul_cox1 <- data.frame("HR" = mul_HR,

"CI95" = mul_CI95,

"P" = mul_PValue)

b=as.data.frame(b)

write.csv(b," Multivariate COX regression.csv")

**mlr3proba-coxph**

# Load the package

library(tidyverse)

library(survival)

library(mlr3verse)

library("devtools")

devtools::install_github("mlr-org/mlr3proba")

library(mlr3proba)

devtools::install_github("mlr-org/mlr3extralearners@*release")

library(mlr3extralearners)

source("tidyfuncs4sa.R")

# file.choose()

# Read the data

gbsg <- readr::read_csv("GBSG.csv")

gbsg <- gbsg[1:23]

colnames(gbsg)

# Correct the variable types.

# Convert categorical variables to factors

for(i in c(1:21)){

gbsg[[i]] <- factor(gbsg[[i]])

}

# Exclude variables - irrelevant variables

gbsg$pid <- NULL

# Exclude samples - samples with missing values, samples with non-positive time.

# gbsg <- na.omit(gbsg) # Exclude samples with missing values in any variable.

gbsg <- gbsg %>%

# na.omit() %>%

drop_na(Age) %>% # Exclude samples with missing values in specified variables.

filter(rfstime > 0) # Exclude samples with non-positive time.

# Data overview.

skimr::skim(gbsg)

DataExplorer::plot_correlation(gbsg)

# Time points of interest.

range(unique(gbsg$rfstime))

itps <- c(365 * c(1, 3, 5))

itps

table(cut(gbsg$rfstime, c(0, itps, Inf)))

###################################################

# Data splitting to construct task objects.

set.seed(42)

datasplit <- rsample::initial_split(

gbsg, prop = 0.8, strata = rfstime, breaks = 10

)

traindata <- rsample::training(datasplit)

testdata <- rsample::testing(datasplit)

# Comparison of survival curves for the split data.

gbsg2 <- gbsg

gbsg2$set <- "test"

gbsg2$set[datasplit$in_id] <- "train"

gbsg2$set <- factor(gbsg2$set)

sfit_set <- survfit(Surv(rfstime, status) ~ set, data=gbsg2)

survminer::ggsurvplot(

sfit_set,

pval=TRUE,

pval.coord = c(0.1, 0.8),

risk.table=TRUE,

ggtheme = theme_minimal()

)

# Data preprocessing.

library(recipes)

datarecipe_coxph <- recipe(rfstime + status ~ ., traindata) %>%

prep()

datarecipe_coxph

# Process the training set and test set accordingly

traindata2 <- bake(datarecipe_coxph, new_data = NULL) %>%

dplyr::select(rfstime, status, everything())

colnames(traindata2)

testdata2 <- bake(datarecipe_coxph, new_data = testdata) %>%

dplyr::select(rfstime, status, everything())

colnames(testdata2)

# The training of the task object

task_train <- as_task_surv(

traindata2,

time = "rfstime",

event = "status",

type = "right"

)

task_train

autoplot(task_train)

# The test of the task object

task_test <- as_task_surv(

testdata2,

time = "rfstime",

event = "status",

type = "right"

)

task_test

autoplot(task_test)

###################################################

# coxph Model

# https://mlr3proba.mlr-org.com/reference/mlr_learners_surv.coxph.html

# Model settings

learner_coxph <- lrn("surv.coxph")

learner_coxph

# Model training

set.seed(42)

learner_coxph$train(task_train)

learner_coxph

# Model overivew

learner_coxph$model

summary(learner_coxph$model)

###################################################

# Predict the training set

predtrain_coxph <- learner_coxph$predict(task_train)

predtrain_coxph

# cindex

predtrain_coxph$score(msrs(c("surv.cindex")))

# Predict the probability of survival at a specified point in time

predprobtrain_coxph <-

predtrain_coxph$distr[

1:nrow(traindata2)

]$survival(itps) %>%

t() %>%

as.data.frame() %>%

mutate(model = "coxph",

dataset = "train",

time = traindata2$rfstime,

status = traindata2$status)

# Evaluation of the model’s performance on the training set

evaltrain_coxph <- eval4sa(

predprob = predprobtrain_coxph,

preddata = traindata2,

etime = "rfstime",

estatus = "status",

model = "coxph",

dataset = "train",

timepoints = itps,

plotcalimethod = "quantile", # nne

bw4nne = NULL,

q4quantile = 5

)

evaltrain_coxph$auc

evaltrain_coxph$roc

evaltrain_coxph$rocplot

evaltrain_coxph$brierscore

evaltrain_coxph$brierscoretest

evaltrain_coxph$calibration

evaltrain_coxph$calibrationplot

# The decision curve at a specified point in time for the training set

tp <- 365*5

tpat <- which(itps == tp)

data.frame(time = traindata2$rfstime,

status = traindata2$status,

Model = 1-predprobtrain_coxph[[tpat]]) %>%

dcurves::dca(

Surv(time, status) ~ Model,

data = .,

time = tp,

label = list(Model = "Coxph"), # Swap Coxph for your own model name

thresholds = 0:100 / 100 # The scope can be changed

) %>%

plot() +

labs(title = "DCA on traindata")

# Predict the test set

predtest_coxph <- learner_coxph$predict(task_test)

predtest_coxph

predtest_coxph$score(msrs(c("surv.cindex")))

# Predict the probability of survival at a specified point in time

predprobtest_coxph <-

predtest_coxph$distr[

1:nrow(testdata2)

]$survival(itps) %>%

t() %>%

as.data.frame() %>%

mutate(model = "coxph",

dataset = "test",

time = testdata2$rfstime,

status = testdata2$status)

# Evaluation of the model’s performance on the test setPredict the test set

evaltest_coxph <- eval4sa(

predprob = predprobtest_coxph,

preddata = testdata2,

etime = "rfstime",

estatus = "status",

model = "coxph",

dataset = "test",

timepoints = itps,

plotcalimethod = "quantile",

bw4nne = NULL,

q4quantile = 3

)

evaltest_coxph$auc

evaltest_coxph$roc

evaltest_coxph$rocplot

evaltest_coxph$brierscore

evaltest_coxph$brierscoretest

evaltest_coxph$calibration

evaltest_coxph$calibrationplot

# The decision curve at a specified point in time for a test set set

tp <- 365*5

tpat <- which(itps == tp)

data.frame(time = testdata2$rfstime,

status = testdata2$status,

Model = 1-predprobtest_coxph[[tpat]])%>%

dcurves::dca(

Surv(time, status) ~ Model,

data = .,

time = tp,

label = list(Model = "Coxph"), # Swap Coxph for your own model name

thresholds = 0:100 / 100 # The scope can be changed

) %>%

plot() +

labs(title = "DCA on testdata")

# Save the results for comparison

save(predtrain_coxph,

predprobtrain_coxph,

evaltrain_coxph,

predtest_coxph,

predprobtest_coxph,

evaltest_coxph,

file = ".\\mlsa\\coxph.RData")

# Save the results for use in the SHINY Web Calculator

traindata4gbsg <- traindata

datarecipe_coxph4gbsg <- datarecipe_coxph

learner_coxph4gbsg <- learner_coxph

save(traindata4gbsg,

datarecipe_coxph4gbsg,

learner_coxph4gbsg,

file = "C:/Construction of machine learning prognostic models/mlsa/coxph.RData")

#############################################

# Model explanation

# The independent variable section

colnames(traindata2)

traindatax <- traindata2[, 3:ncol(traindata2)]

colnames(traindatax)

# Interpreter – Based on the training set, no point in time can be specified

exper_coxph <- survex::explain(

learner_coxph$model,

data = traindatax,

y = survival::Surv(

time = traindata2$rfstime,

event = traindata2$status

),

times = itps

)

# Variable importance

set.seed(42)

vip_coxph <- survex::model_parts(

exper_coxph,

type = "ratio",

N = 100

)

plot(vip_coxph, max_vars = ncol(traindatax)+1)

# Variable importance bar chart of tick points

vip_coxph$result %>%

filter(`_permutation_` == 0) %>%

rename("Time" = "_times_") %>%

select(Time, all_of(colnames(traindatax))) %>%

pivot_longer(cols = -1) %>%

mutate(name = tidytext::reorder_within(name, value, Time)) %>%

ggplot(aes(x = value, y = name)) +

geom_col(fill = "skyblue") +

geom_vline(xintercept = 1) +

tidytext::scale_y_reordered() +

labs(x = "importance", y = "") +

facet_wrap(~Time, scales = "free_y", labeller = "label_both") +

theme_minimal()

# Partial dependency graphs

set.seed(42)

partial_coxph <- survex::model_profile(

exper_coxph,

variables = c("pgr", "hormon"),

# Specified as a categorical variable

categorical_variables = c("hormon"),

N = 100,

grid_points = 11

)

plot(partial_coxph, facet_ncol = 1)

# Single-shot prediction decomposition

set.seed(42)

shap_coxph <- survex::predict_parts(

exper_coxph,

new_observation = traindatax[1, ], # 1st sample

N = 100,

calculation_method = "kernelshap"

)

plot(shap_coxph, max_vars = ncol(traindatax))

# SHAP of all samples

shaps_coxph_df <- traindatax %>%

pmap_dfr(

function(...) {

current <- tibble(...)

set.seed(42)

shapi_coxph <- survex::predict_parts(

exper_coxph,

new_observation = current,

N = 100,

calculation_method = "kernelshap"

)

return(shapi_coxph$result %>% rownames_to_column("Time"))

}

)

shaps_coxph_df2 <- shaps_coxph_df %>%

mutate(id = rep(1:nrow(traindatax), each = length(itps))) %>%

select(id, Time, everything()) %>%

pivot_longer(cols = -c(1,2), values_to = "shapley")

shapleyimp_coxph <- shaps_coxph_df2 %>%

group_by(name) %>%

summarise(shapley.abs.mean = mean(abs(shapley), na.rm = T)) %>%

arrange(shapley.abs.mean) %>%

mutate(name = as_factor(name))

flname <- colnames(traindatax)[c(2,4,8)]

lxname <- colnames(traindatax)[-c(2,4,8)]

library(ggh4x)

traindatax %>%

mutate(id = 1:n()) %>%

select(id, all_of(flname)) %>%

pivot_longer(cols = -1) %>%

left_join(shaps_coxph_df2, by = c("id", "name")) %>%

mutate(Time = as_factor(Time)) %>%

ggplot(aes(x = interaction(value, name), y = shapley)) +

geom_boxplot(aes(fill = name), show.legend = F) +

geom_hline(yintercept = 0, color = "grey10") +

scale_x_discrete(NULL, guide = "axis_nested") +

scale_colour_viridis_c() +

labs(x = "", y = "Shapley") +

facet_wrap(~Time, ncol = 1, scales = "free") +

theme_bw() +

theme(axis.text.x = element_text(angle = 30, hjust = 1),

legend.position = "bottom")

traindatax %>%

mutate(id = 1:n()) %>%

select(id, all_of(lxname)) %>%

pivot_longer(cols = -1) %>%

left_join(shaps_coxph_df2, by = c("id", "name")) %>%

mutate(Time = as_factor(Time)) %>%

dplyr::group_by(name) %>%

dplyr::mutate(

value = (value - min(value)) / (max(value) - min(value)),

name = factor(name, levels = levels(shapleyimp_coxph$name))

) %>%

dplyr::arrange(value) %>%

dplyr::ungroup() %>%

ggplot(aes(x = shapley, y = name, color = value)) +

ggbeeswarm::geom_quasirandom(width = 0.2) +

scale_color_gradient(

low = "red",

high = "blue",

breaks = c(0, 1),

labels = c("Low", "High"),

guide = guide_colorbar(barwidth = 0.5,

barheight = length(lxname)*2,

ticks = F,

title.position = "right",

title.hjust = 0.5)

) +

labs(x = "Shapley", y = "Feature", color = "Feature value") +

facet_wrap(~Time) +

theme_bw() +

theme(legend.title = element_text(angle = -90))

**mlr3proba-lasso**

# Load the package

library(tidyverse)

library(survival)

library(mlr3verse)

# remotes::install_github("mlr-org/mlr3proba")

library(mlr3proba)

# remotes::install_github("mlr-org/mlr3extralearners@*release")

library(mlr3extralearners)

source("tidyfuncs4sa.R")

# file.choose()

# Read the data

gbsg <- readr::read_csv("data.csv")

gbsg=gbsg[,1:23]

colnames(gbsg)

# Correct the variable types.

# Convert categorical variables to factors

for(i in c(1:21)){

gbsg[[i]] <- factor(gbsg[[i]])

}

# Exclude variables - irrelevant variables

gbsg$pid <- NULL

# Exclude samples - samples with missing values, samples with non-positive time

# gbsg <- na.omit(gbsg) # Exclude samples with missing values in any variable

gbsg <- gbsg %>%

drop_na(age) %>% # Exclude samples with missing values in specified variables

filter(rfstime > 0) # Exclude samples with non-positive time

# Data overview.

skimr::skim(gbsg)

DataExplorer::plot_correlation(gbsg)

# Time points of interest

sort(unique(gbsg$rfstime))

itps <- c(365 * c(1, 3, 5))

itps

table(cut(gbsg$rfstime, c(0, itps, Inf)))

###################################################

# Data splitting to construct task objects

set.seed(42)

datasplit <- rsample::initial_split(

gbsg, prop = 0.8, strata = rfstime, breaks = 10

)

traindata <- rsample::training(datasplit)

testdata <- rsample::testing(datasplit)

# Comparison of survival curves for the split data

gbsg2 <- gbsg

gbsg2$set <- "test"

gbsg2$set[datasplit$in_id] <- "train"

gbsg2$set <- factor(gbsg2$set)

sfit_set <- survfit(Surv(rfstime, status) ~ set, data=gbsg2)

survminer::ggsurvplot(

sfit_set,

pval=TRUE,

pval.coord = c(0.1, 0.8),

risk.table=TRUE,

ggtheme = theme_minimal()

)

# Data preprocessing

library(recipes)

datarecipe_lasso <- recipe(rfstime + status ~ ., traindata) %>%

step_dummy(all_nominal_predictors()) %>%

prep()

datarecipe_lasso

# Process the training set and test set accordingly

traindata2 <- bake(datarecipe_lasso, new_data = NULL) %>%

dplyr::select(rfstime, status, everything())

colnames(traindata2)

testdata2 <- bake(datarecipe_lasso, new_data = testdata) %>%

dplyr::select(rfstime, status, everything())

colnames(testdata2)

# The training of the task object

task_train <- as_task_surv(

traindata2,

time = "rfstime",

event = "status",

type = "right"

)

task_train

# The test of the task object

task_test <- as_task_surv(

testdata2,

time = "rfstime",

event = "status",

type = "right"

)

task_test

###################################################

# lasso Model

# https://mlr3extralearners.mlr-org.com/reference/mlr_learners_surv.cv_glmnet.html

# Model settings

learner_lasso_0 <- lrn(

"surv.cv_glmnet",

type.measure = "C",

s = "lambda.1se"

)

learner_lasso_0

learner_lasso_1 <- ppl(

"distrcompositor",

learner = learner_lasso_0,

estimator = "kaplan",

form = "ph"

)

learner_lasso_1

# learner_lasso_1$plot()

learner_lasso <- as_learner(

learner_lasso_1,

)

learner_lasso

# Model training

set.seed(42)

learner_lasso$train(task_train)

learner_lasso

# Model overivew

learner_lasso$model$surv.cv_glmnet$model

coef(learner_lasso$model$surv.cv_glmnet$model)

plot(learner_lasso$model$surv.cv_glmnet$model)

###################################################

# Predict the training set

predtrain_lasso <- learner_lasso$predict(task_train)

predtrain_lasso

# cindex

predtrain_lasso$score(msrs(c("surv.cindex")))

# Predict the probability of survival at a specified point in time

predprobtrain_lasso <-

predtrain_lasso$distr[

1:nrow(traindata2)

]$survival(itps) %>%

t() %>%

as.data.frame() %>%

mutate(model = "lasso",

dataset = "train",

time = traindata2$rfstime,

status = traindata2$status)

# Evaluation of the model’s performance on the training set

evaltrain_lasso <- eval4sa(

predprob = predprobtrain_lasso,

preddata = traindata2,

etime = "rfstime",

estatus = "status",

model = "lasso",

dataset = "train",

timepoints = itps,

plotcalimethod = "quantile",

bw4nne = NULL,

q4quantile = 5

)

evaltrain_lasso$auc

evaltrain_lasso$roc

evaltrain_lasso$rocplot

evaltrain_lasso$brierscore

evaltrain_lasso$brierscoretest

evaltrain_lasso$calibration

evaltrain_lasso$calibrationplot

# The decision curve at a specified point in time for the training set

tp <- 365*5

tpat <- which(itps == tp)

data.frame(time = traindata2$rfstime,

status = traindata2$status,

Model = 1-predprobtrain_lasso[[tpat]])%>%

dcurves::dca(

Surv(time, status) ~ Model,

data = .,

time = tp,

label = list(Model = "lasso"), # Lasso can be replaced with its own model name

thresholds = 0:100 / 100 # The scope can be changed

) %>%

plot() +

labs(title = "DCA on traindata")

# Predict the test set

predtest_lasso <- learner_lasso$predict(task_test)

predtest_lasso

predtest_lasso$score(msrs(c("surv.cindex")))

# Predict the probability of survival at a specified point in time

predprobtest_lasso <-

predtest_lasso$distr[

1:nrow(testdata2)

]$survival(itps) %>%

t() %>%

as.data.frame() %>%

mutate(model = "lasso",

dataset = "test",

time = testdata2$rfstime,

status = testdata2$status)

# Evaluation of the model’s performance on the test setPredict the test set

evaltest_lasso <- eval4sa(

predprob = predprobtest_lasso,

preddata = testdata2,

etime = "rfstime",

estatus = "status",

model = "lasso",

dataset = "test",

timepoints = itps,

plotcalimethod = "quantile",

bw4nne = NULL,

q4quantile = 3

)

evaltest_lasso$auc

evaltest_lasso$roc

evaltest_lasso$rocplot

evaltest_lasso$brierscore

evaltest_lasso$brierscoretest

evaltest_lasso$calibration

evaltest_lasso$calibrationplot

# The decision curve at a specified point in time for a test set set

tp <- 365*5

tpat <- which(itps == tp)

data.frame(time = testdata2$rfstime,

status = testdata2$status,

Model = 1-predprobtest_lasso[[tpat]])%>%

dcurves::dca(

Surv(time, status) ~ Model,

data = .,

time = tp,

label = list(Model = "lasso"), # Lasso can be replaced with its own model name

thresholds = 0:100 / 100 # The scope can be changed

) %>%

plot() +

labs(title = "DCA on testdata")

# Save the results for comparison

save(predtrain_lasso,

predprobtrain_lasso,

evaltrain_lasso,

predtest_lasso,

predprobtest_lasso,

evaltest_lasso,

file = ".\\mlsa\\lasso.RData")

# Save the results for use in the SHINY Web Calculator

traindata4gbsg <- traindata

datarecipe_lasso4gbsg <- datarecipe_lasso

learner_lasso4gbsg <- learner_lasso

save(traindata4gbsg,

datarecipe_lasso4gbsg,

learner_lasso4gbsg,

file = ".\\mlsashiny\\lasso.RData")

#############################################

# Model explanation

# The independent variable section

colnames(traindata2)

traindatax <- traindata2[, 3:ncol(traindata2)]

colnames(traindatax)

# Interpreter – Based on the training set, no point in time can be specified

exper_lasso <- survex::explain_survival(

learner_lasso,

data = traindatax,

y = survival::Surv(

time = traindata2$rfstime,

event = traindata2$status

),

predict_function = risk_pred,

predict_survival_function = surv_pred,

predict_cumulative_hazard_function = chf_pred,

label = "lasso",

times = itps

)

# Variable importance

set.seed(42)

vip_lasso <- survex::model_parts(

exper_lasso,

type = "ratio",

N = 100

)

plot(vip_lasso, max_vars = ncol(traindatax)+1)

# Variable importance bar chart of tick points

vip_lasso$result %>%

filter(`_permutation_` == 0) %>%

rename("Time" = "_times_") %>%

select(Time, all_of(colnames(traindatax))) %>%

pivot_longer(cols = -1) %>%

mutate(name = tidytext::reorder_within(name, value, Time)) %>%

ggplot(aes(x = value, y = name)) +

geom_col(fill = "skyblue") +

geom_vline(xintercept = 1) +

tidytext::scale_y_reordered() +

labs(x = "importance", y = "") +

facet_wrap(~Time, scales = "free_y", labeller = "label_both") +

theme_minimal()

# Partial dependency graphs

set.seed(42)

partial_lasso <- survex::model_profile(

exper_lasso,

variables = c("pgr", "hormon_X1"),

# Specified as a categorical variable

categorical_variables = c("hormon_X1"),

N = 100,

grid_points = 11

)

plot(partial_lasso, facet_ncol = 1)

# Single-shot prediction decomposition

set.seed(42)

shap_lasso <- survex::predict_parts(

exper_lasso,

new_observation = traindatax[1, ], # 1st sample

N = 100,

calculation_method = "kernelshap"

)

plot(shap_lasso, max_vars = ncol(traindatax))

# SHAP of all samples

shaps_lasso_df <- traindatax %>%

pmap_dfr(

function(...) {

current <- tibble(...)

set.seed(42)

shapi_lasso <- survex::predict_parts(

exper_lasso,

new_observation = current,

N = 100,

calculation_method = "kernelshap"

)

return(shapi_lasso$result %>% rownames_to_column("Time"))

}

)

shaps_lasso_df2 <- shaps_lasso_df %>%

mutate(id = rep(1:nrow(traindatax), each = length(itps))) %>%

select(id, Time, everything()) %>%

pivot_longer(cols = -c(1,2), values_to = "shapley")

shapleyimp_lasso <- shaps_lasso_df2 %>%

group_by(name) %>%

summarise(shapley.abs.mean = mean(abs(shapley), na.rm = T)) %>%

arrange(shapley.abs.mean) %>%

mutate(name = as_factor(name))

flname <- colnames(traindatax)[c(2,4,8)]

lxname <- colnames(traindatax)[-c(2,4,8)]

library(ggh4x)

traindatax %>%

mutate(id = 1:n()) %>%

select(id, all_of(flname)) %>%

pivot_longer(cols = -1) %>%

left_join(shaps_lasso_df2, by = c("id", "name")) %>%

mutate(Time = as_factor(Time)) %>%

ggplot(aes(x = interaction(value, name), y = shapley)) +

geom_boxplot(aes(fill = name), show.legend = F) +

geom_hline(yintercept = 0, color = "grey10") +

scale_x_discrete(NULL, guide = "axis_nested") +

scale_colour_viridis_c() +

labs(x = "") +

facet_wrap(~Time, ncol = 1, scales = "free_y") +

theme_bw() +

theme(axis.text.x = element_text(angle = 30, hjust = 1),

legend.position = "bottom")

traindatax %>%

mutate(id = 1:n()) %>%

select(id, all_of(lxname)) %>%

pivot_longer(cols = -1) %>%

left_join(shaps_lasso_df2, by = c("id", "name")) %>%

mutate(Time = as_factor(Time)) %>%

dplyr::group_by(name) %>%

dplyr::mutate(

value = (value - min(value)) / (max(value) - min(value)),

name = factor(name, levels = levels(shapleyimp_lasso$name))

) %>%

dplyr::arrange(value) %>%

dplyr::ungroup() %>%

ggplot(aes(x = shapley, y = name, color = value)) +

ggbeeswarm::geom_quasirandom(width = 0.2) +

scale_color_gradient(

low = "red",

high = "blue",

breaks = c(0, 1),

labels = c("Low", "High"),

guide = guide_colorbar(barwidth = 0.5,

barheight = length(lxname)*2,

ticks = F,

title.position = "right",

title.hjust = 0.5)

) +

labs(x = "SHAP value", color = "Feature value") +

facet_wrap(~Time) +

theme_bw() +

theme(legend.title = element_text(angle = -90))

**mlr3proba-decision tree**

# Load the package

library(tidyverse)

library(survival)

library(mlr3verse)

# remotes::install_github("mlr-org/mlr3proba")

library(mlr3proba)

# remotes::install_github("mlr-org/mlr3extralearners@*release")

library(mlr3extralearners)

source("tidyfuncs4sa.R")

# file.choose()

# Read the data

gbsg <- readr::read_csv("GBSG.csv")

colnames(gbsg)

# Correct the variable types.

# Convert categorical variables to factors

for(i in c(3, 5, 9)){

gbsg[[i]] <- factor(gbsg[[i]])

}

# Exclude variables - irrelevant variables

gbsg$pid <- NULL

# Exclude samples - samples with missing values, samples with non-positive time

# gbsg <- na.omit(gbsg) # Exclude samples with missing values in any variable

gbsg <- gbsg %>%

drop_na(age) %>% # Exclude samples with missing values in specified variables

filter(rfstime > 0) # Exclude samples with non-positive time

# Data overview.

skimr::skim(gbsg)

DataExplorer::plot_correlation(gbsg)

# Time points of interest

sort(unique(gbsg$rfstime))

itps <- c(365 * c(1, 3, 5))

itps

table(cut(gbsg$rfstime, c(0, itps, Inf)))

###################################################

# Data splitting to construct task objects

set.seed(42)

datasplit <- rsample::initial_split(

gbsg, prop = 0.8, strata = rfstime, breaks = 10

)

traindata <- rsample::training(datasplit)

testdata <- rsample::testing(datasplit)

# Comparison of survival curves for the split data

gbsg2 <- gbsg

gbsg2$set <- "test"

gbsg2$set[datasplit$in_id] <- "train"

gbsg2$set <- factor(gbsg2$set)

sfit_set <- survfit(Surv(rfstime, status) ~ set, data=gbsg2)

survminer::ggsurvplot(

sfit_set,

pval=TRUE,

pval.coord = c(0.1, 0.8),

risk.table=TRUE,

ggtheme = theme_minimal()

)

# Data preprocessing

library(recipes)

datarecipe_rpart <- recipe(rfstime + status ~ ., traindata) %>%

prep()

datarecipe_rpart

# Process the training set and test set accordingly

traindata2 <- bake(datarecipe_rpart, new_data = NULL) %>%

dplyr::select(rfstime, status, everything())

colnames(traindata2)

testdata2 <- bake(datarecipe_rpart, new_data = testdata) %>%

dplyr::select(rfstime, status, everything())

colnames(testdata2)

# The training of the task object

task_train <- as_task_surv(

traindata2,

time = "rfstime",

event = "status",

type = "right"

)

task_train

# The test of the task object

task_test <- as_task_surv(

testdata2,

time = "rfstime",

event = "status",

type = "right"

)

task_test

###################################################

# Conditional Inference Tree

# https://mlr3proba.mlr-org.com/reference/mlr_learners_surv.rpart.html

# Set up the model

learner_rpart_0 <- ppl(

"distrcompositor",

learner = lrn(

"surv.rpart",

cp = to_tune(0.001, 0.5),

minbucket = to_tune(5, 9)

),

estimator = "kaplan",

form = "ph"

) %>%

as_learner()

learner_rpart_0

# Hyperparameter tuning settings

learner_rpart <- auto_tuner(

tuner = tnr(

"grid_search",

param_resolutions = c(surv.rpart.cp = 5,

surv.rpart.minbucket = 3),

batch_size = 4

),

learner = learner_rpart_0,

resampling = rsmp("cv", folds = 5),

measure = msr("surv.cindex"),

terminator = trm("none")

)

learner_rpart

# Multi-core parallelism

future::plan("multisession")

Training

set.seed(42)

learner_rpart$train(task_train)

# Model overivew

learner_rpart

learner_rpart$tuning_result

learner_rpart$tuning_instance

autoplot(learner_rpart$tuning_instance)

# learner_rpart$tuning_instance$archive

# learner_rpart$tuning_instance$result_learner_param_vals

learner_rpart$learner$model

###################################################

# Predict the training set

predtrain_rpart <- learner_rpart$predict(task_train)

predtrain_rpart

# cindex

predtrain_rpart$score(msrs(c("surv.cindex")))

# Predict the probability of survival at a specified point in time

predprobtrain_rpart <-

predtrain_rpart$distr[

1:nrow(traindata2)

]$survival(itps) %>%

t() %>%

as.data.frame() %>%

mutate(model = "rpart",

dataset = "train",

time = traindata2$rfstime,

status = traindata2$status)

colnames(predprobtrain_rpart)[1:length(itps)] <- as.character(itps)

# Evaluation of the model’s performance on the training set

evaltrain_rpart <- eval4sa(

predprob = predprobtrain_rpart,

preddata = traindata2,

etime = "rfstime",

estatus = "status",

model = "rpart",

dataset = "train",

timepoints = itps,

plotcalimethod = "quantile",

bw4nne = NULL,

q4quantile = 3

)

evaltrain_rpart$auc

evaltrain_rpart$roc

evaltrain_rpart$rocplot

evaltrain_rpart$brierscore

evaltrain_rpart$brierscoretest

evaltrain_rpart$calibration

evaltrain_rpart$calibrationplot

# The decision curve at a specified point in time for the training set

tp <- 365*5

tpat <- which(itps == tp)

data.frame(time = traindata2$rfstime,

status = traindata2$status,

Model = 1-predprobtrain_rpart[[tpat]])%>%

dcurves::dca(

Surv(time, status) ~ Model,

data = .,

time = tp,

label = list(Model = "rpart"), # rpart can be replaced with your own model name

thresholds = 0:100 / 100 # The scope can be changed

) %>%

plot() +

labs(title = "DCA on traindata")

# Predict the test set

predtest_rpart <- learner_rpart$predict(task_test)

predtest_rpart

predtest_rpart$score(msrs(c("surv.cindex")))

# Predict the probability of survival at a specified point in time

predprobtest_rpart <-

predtest_rpart$distr[

1:nrow(testdata2)

]$survival(itps) %>%

t() %>%

as.data.frame() %>%

mutate(model = "rpart",

dataset = "test",

time = testdata2$rfstime,

status = testdata2$status)

colnames(predprobtest_rpart)[1:length(itps)] <- as.character(itps)

# Evaluation of the model’s performance on the test setPredict the test set

evaltest_rpart <- eval4sa(

predprob = predprobtest_rpart,

preddata = testdata2,

etime = "rfstime",

estatus = "status",

model = "rpart",

dataset = "test",

timepoints = itps,

plotcalimethod = "quantile",

bw4nne = NULL,

q4quantile = 3

)

evaltest_rpart$auc

evaltest_rpart$roc

evaltest_rpart$rocplot

evaltest_rpart$brierscore

evaltest_rpart$brierscoretest

evaltest_rpart$calibration

evaltest_rpart$calibrationplot

# The decision curve at a specified point in time for a test set set

tp <- 365*5

tpat <- which(itps == tp)

data.frame(time = testdata2$rfstime,

status = testdata2$status,

Model = 1-predprobtest_rpart[[tpat]])%>%

dcurves::dca(

Surv(time, status) ~ Model,

data = .,

time = tp,

label = list(Model = "rpart"), # ctree can be replaced with your own model name

thresholds = 0:100 / 100 # The scope can be changed

) %>%

plot() +

labs(title = "DCA on testdata")

# Save the results for comparison

save(predtrain_rpart,

predprobtrain_rpart,

evaltrain_rpart,

predtest_rpart,

predprobtest_rpart,

evaltest_rpart,

file = ".\\mlsa\\ctree.RData")

# Save the results for use in the SHINY Web Calculator

traindata4gbsg <- traindata

datarecipe_rpart4gbsg <- datarecipe_rpart

learner_rpart4gbsg <- learner_rpart

save(traindata4gbsg,

datarecipe_rpart4gbsg,

learner_rpart4gbsg,

file = ".\\mlsashiny\\ctree.RData")

#############################################

# Model explanation

# The independent variable section

colnames(traindata2)

traindatax <- traindata2[, 3:ncol(traindata2)]

colnames(traindatax)

# Interpreter – Based on the training set, no point in time can be specified

exper_rpart <- survex::explain_survival(

learner_rpart,

data = traindatax,

y = survival::Surv(

time = traindata2$rfstime,

event = traindata2$status

),

predict_function = risk_pred,

predict_survival_function = surv_pred,

predict_cumulative_hazard_function = chf_pred,

label = "rpart",

times = itps

)

# Variable importance

set.seed(42)

vip_rpart <- survex::model_parts(

exper_rpart,

type = "ratio",

N = 100

)

plot(vip_rpart, max_vars = ncol(traindatax)+1, subtitle = NULL)

# Variable importance bar chart of tick points

vip_rpart$result %>%

filter(`_permutation_` == 0) %>%

rename("Time" = "_times_") %>%

select(Time, all_of(colnames(traindatax))) %>%

pivot_longer(cols = -1) %>%

mutate(name = tidytext::reorder_within(name, value, Time)) %>%

ggplot(aes(x = value, y = name)) +

geom_col(fill = "skyblue") +

geom_vline(xintercept = 1) +

tidytext::scale_y_reordered() +

labs(x = "importance", y = "") +

facet_wrap(~Time, scales = "free_y", labeller = "label_both") +

theme_minimal()

# Partial dependency graphs

set.seed(42)

partial_rpart <- survex::model_profile(

exper_rpart,

variables = c("pgr", "hormon"),

# Specified as a categorical variable

categorical_variables = c("hormon"),

N = 100,

grid_points = 11

)

plot(partial_rpart, facet_ncol = 1, subtitle = NULL)

# Single-shot prediction decomposition

set.seed(42)

shap_rpart <- survex::predict_parts(

exper_rpart,

new_observation = traindatax[1, ], # 1st sample

N = 100,

calculation_method = "kernelshap"

)

plot(shap_rpart, max_vars = ncol(traindatax), subtitle = NULL)

# SHAP of all samples

shaps_rpart_df <- traindatax %>%

pmap_dfr(

function(...) {

current <- tibble(...)

set.seed(42)

shapi_rpart <- survex::predict_parts(

exper_rpart,

new_observation = current,

N = 100,

calculation_method = "kernelshap"

)

return(shapi_rpart$result %>% rownames_to_column("Time"))

}

)

shaps_rpart_df2 <- shaps_rpart_df %>%

mutate(id = rep(1:nrow(traindatax), each = length(itps))) %>%

select(id, Time, everything()) %>%

pivot_longer(cols = -c(1,2), values_to = "shapley")

shapleyimp_rpart <- shaps_rpart_df2 %>%

group_by(name) %>%

summarise(shapley.abs.mean = mean(abs(shapley), na.rm = T)) %>%

arrange(shapley.abs.mean) %>%

mutate(name = as_factor(name))

flname <- colnames(traindatax)[c(2,4,8)]

lxname <- colnames(traindatax)[-c(2,4,8)]

library(ggh4x)

traindatax %>%

mutate(id = 1:n()) %>%

select(id, all_of(flname)) %>%

pivot_longer(cols = -1) %>%

left_join(shaps_rpart_df2, by = c("id", "name")) %>%

mutate(Time = as_factor(Time)) %>%

ggplot(aes(x = interaction(value, name), y = shapley)) +

geom_boxplot(aes(fill = name), show.legend = F) +

geom_hline(yintercept = 0, color = "grey10") +

scale_x_discrete(NULL, guide = "axis_nested") +

scale_colour_viridis_c() +

labs(x = "") +

facet_wrap(~Time, ncol = 1, scales = "free_y") +

theme_bw() +

theme(axis.text.x = element_text(angle = 30, hjust = 1),

legend.position = "bottom")

traindatax %>%

mutate(id = 1:n()) %>%

select(id, all_of(lxname)) %>%

pivot_longer(cols = -1) %>%

left_join(shaps_rpart_df2, by = c("id", "name")) %>%

mutate(Time = as_factor(Time)) %>%

dplyr::group_by(name) %>%

dplyr::mutate(

value = (value - min(value)) / (max(value) - min(value)),

name = factor(name, levels = levels(shapleyimp_rpart$name))

) %>%

dplyr::arrange(value) %>%

dplyr::ungroup() %>%

ggplot(aes(x = shapley, y = name, color = value)) +

ggbeeswarm::geom_quasirandom(width = 0.2) +

scale_color_gradient(

low = "red",

high = "blue",

breaks = c(0, 1),

labels = c("Low", "High"),

guide = guide_colorbar(barwidth = 0.5,

barheight = length(lxname)*2,

ticks = F,

title.position = "right",

title.hjust = 0.5)

) +

labs(x = "SHAP value", color = "Feature value") +

facet_wrap(~Time) +

theme_bw() +

theme(legend.title = element_text(angle = -90))

**mlr3proba-Random forest**

# Load the package

library(tidyverse)

library(survival)

library(mlr3verse)

# remotes::install_github("mlr-org/mlr3proba")

library(mlr3proba)

# remotes::install_github("mlr-org/mlr3extralearners@*release")

library(mlr3extralearners)

source("tidyfuncs4sa.R")

# file.choose()

# Read the data

gbsg <- readr::read_csv("GBSG.csv")

colnames(gbsg)

# Correct the variable types.

# Convert categorical variables to factors

for(i in c(3, 5, 9)){

gbsg[[i]] <- factor(gbsg[[i]])

}

# Exclude variables - irrelevant variables

gbsg$pid <- NULL

# Exclude samples - samples with missing values, samples with non-positive time

# gbsg <- na.omit(gbsg) # Exclude samples with missing values in any variable

gbsg <- gbsg %>%

drop_na(age) %>% # Exclude samples with missing values in specified variables

filter(rfstime > 0) # Exclude samples with non-positive time

# Data overview.

skimr::skim(gbsg)

DataExplorer::plot_correlation(gbsg)

# Time points of interest

sort(unique(gbsg$rfstime))

itps <- c(365 * c(1, 3, 5))

itps

table(cut(gbsg$rfstime, c(0, itps, Inf)))

###################################################

# Data splitting to construct task objects

set.seed()

datasplit <- rsample::initial_split(

gbsg, prop = 0.8, strata = rfstime, breaks = 10

)

traindata <- rsample::training(datasplit)

testdata <- rsample::testing(datasplit)

# Comparison of survival curves for the split data

gbsg2 <- gbsg

gbsg2$set <- "test"

gbsg2$set[datasplit$in_id] <- "train"

gbsg2$set <- factor(gbsg2$set)

sfit_set <- survfit(Surv(rfstime, status) ~ set, data=gbsg2)

survminer::ggsurvplot(

sfit_set,

pval=TRUE,

pval.coord = c(0.1, 0.8),

risk.table=TRUE,

ggtheme = theme_minimal()

)

# Data preprocessing

library(recipes)

datarecipe_rsf <- recipe(rfstime + status ~ ., traindata) %>%

prep()

datarecipe_rsf

# Process the training set and test set accordingly

traindata2 <- bake(datarecipe_rsf, new_data = NULL) %>%

dplyr::select(rfstime, status, everything())

colnames(traindata2)

testdata2 <- bake(datarecipe_rsf, new_data = testdata) %>%

dplyr::select(rfstime, status, everything())

colnames(testdata2)

# The training of the task object

task_train <- as_task_surv(

traindata2,

time = "rfstime",

event = "status",

type = "right"

)

task_train

# The test of the task object

task_test <- as_task_surv(

testdata2,

time = "rfstime",

event = "status",

type = "right"

)

task_test

###################################################

# Random forest

# https://mlr3extralearners.mlr-org.com/reference/mlr_learners_surv.rfsrc.html

# Set up the model

learner_rsf_0 <- lrn(

"surv.rfsrc",

ntree = to_tune(200, 500),

mtry = to_tune(3, 5),

nodesize = to_tune(15, 21),

predict_type = "distr"

)

learner_rsf_0

# Hyperparameter tuning settings

learner_rsf <- auto_tuner(

tuner = tnr("grid_search", resolution = 3, batch_size = 3),

learner = learner_rsf_0,

resampling = rsmp("cv", folds = 5),

measure = msr("surv.cindex"),

terminator = trm("none")

)

learner_rsf

# Multi-core parallelism

future::plan("multisession")

Training

set.seed(42)

learner_rsf$train(task_train)

# Model overivew

learner_rsf

learner_rsf$tuning_result

learner_rsf$tuning_instance

autoplot(learner_rsf$tuning_instance)

# learner_rsf$tuning_instance$archive

# learner_rsf$tuning_instance$result_learner_param_vals

learner_rsf$learner$model

###################################################

# Predict the training set

predtrain_rsf <- learner_rsf$predict(task_train)

predtrain_rsf

# cindex

predtrain_rsf$score(msrs(c("surv.cindex")))

# Predict the probability of survival at a specified point in time

predprobtrain_rsf <-

predtrain_rsf$distr[

1:nrow(traindata2)

]$survival(itps) %>%

t() %>%

as.data.frame() %>%

mutate(model = "rsf",

dataset = "train",

time = traindata2$rfstime,

status = traindata2$status)

# Evaluation of the model’s performance on the training set

evaltrain_rsf <- eval4sa(

predprob = predprobtrain_rsf,

preddata = traindata2,

etime = "rfstime",

estatus = "status",

model = "rsf",

dataset = "train",

timepoints = itps,

plotcalimethod = "quantile",

bw4nne = NULL,

q4quantile = 5

)

evaltrain_rsf$auc

evaltrain_rsf$roc

evaltrain_rsf$rocplot

evaltrain_rsf$brierscore

evaltrain_rsf$brierscoretest

evaltrain_rsf$calibration

evaltrain_rsf$calibrationplot

# The decision curve at a specified point in time for the training set

tp <- 365*5

tpat <- which(itps == tp)

data.frame(time = traindata2$rfstime,

status = traindata2$status,

Model = 1-predprobtrain_rsf[[tpat]])%>%

dcurves::dca(

Surv(time, status) ~ Model,

data = .,

time = tp,

label = list(Model = "rsf"), # rsf can be replaced with its own model name

thresholds = 0:100 / 100 # The scope can be changed

) %>%

plot() +

labs(title = "DCA on traindata")

# Predict the test set

predtest_rsf <- learner_rsf$predict(task_test)

predtest_rsf

predtest_rsf$score(msrs(c("surv.cindex")))

# Predict the probability of survival at a specified point in time

predprobtest_rsf <-

predtest_rsf$distr[

1:nrow(testdata2)

]$survival(itps) %>%

t() %>%

as.data.frame() %>%

mutate(model = "rsf",

dataset = "test",

time = testdata2$rfstime,

status = testdata2$status)

# Evaluation of the model’s performance on the test setPredict the test set

evaltest_rsf <- eval4sa(

predprob = predprobtest_rsf,

preddata = testdata2,

etime = "rfstime",

estatus = "status",

model = "rsf",

dataset = "test",

timepoints = itps,

plotcalimethod = "quantile",

bw4nne = NULL,

q4quantile = 3

)

evaltest_rsf$auc

evaltest_rsf$roc

evaltest_rsf$rocplot

evaltest_rsf$brierscore

evaltest_rsf$brierscoretest

evaltest_rsf$calibration

evaltest_rsf$calibrationplot

# The decision curve at a specified point in time for a test set set

tp <- 365*5

tpat <- which(itps == tp)

data.frame(time = testdata2$rfstime,

status = testdata2$status,

Model = 1-predprobtest_rsf[[tpat]])%>%

dcurves::dca(

Surv(time, status) ~ Model,

data = .,

time = tp,

label = list(Model = "rsf"), # rsf can be replaced with its own model name

thresholds = 0:100 / 100 # The scope can be changed

) %>%

plot() +

labs(title = "DCA on testdata")

# Save the results for comparison

save(predtrain_rsf,

predprobtrain_rsf,

evaltrain_rsf,

predtest_rsf,

predprobtest_rsf,

evaltest_rsf,

file = ".\\mlsa\\rsf.RData")

# Save the results for use in the SHINY Web Calculator

traindata4gbsg <- traindata

datarecipe_rsf4gbsg <- datarecipe_rsf

learner_rsf4gbsg <- learner_rsf

save(traindata4gbsg,

datarecipe_rsf4gbsg,

learner_rsf4gbsg,

file = ".\\mlsashiny\\rsf.RData")

#############################################

# Model explanation

# The independent variable section

colnames(traindata2)

traindatax <- traindata2[, 3:ncol(traindata2)]

colnames(traindatax)

# Interpreter – Based on the training set, no point in time can be specified

exper_rsf <- survex::explain(

learner_rsf$learner$model,

data = traindatax,

y = survival::Surv(

time = traindata2$rfstime,

event = traindata2$status

),

times = itps

)

# Variable importance

set.seed()

vip_rsf <- survex::model_parts(

exper_rsf,

type = "ratio",

N = 100

)

plot(vip_rsf, max_vars = ncol(traindatax)+1)

# Variable importance bar chart of tick points

vip_rsf$result %>%

filter(`_permutation_` == 0) %>%

rename("Time" = "_times_") %>%

select(Time, all_of(colnames(traindatax))) %>%

pivot_longer(cols = -1) %>%

mutate(name = tidytext::reorder_within(name, value, Time)) %>%

ggplot(aes(x = value, y = name)) +

geom_col(fill = "skyblue") +

geom_vline(xintercept = 1) +

tidytext::scale_y_reordered() +

labs(x = "importance", y = "") +

facet_wrap(~Time, scales = "free_y", labeller = "label_both") +

theme_minimal()

# Partial dependency graphs

set.seed()

partial_rsf <- survex::model_profile(

exper_rsf,

variables = c("pgr", "hormon"),

# Specified as a categorical variable

categorical_variables = c("hormon"),

N = 100,

grid_points = 11

)

plot(partial_rsf, facet_ncol = 1)

# Single-shot prediction decomposition

set.seed()

shap_rsf <- survex::predict_parts(

exper_rsf,

new_observation = traindatax[1, ], # 1st sample

N = 100,

calculation_method = "kernelshap"

)

plot(shap_rsf, max_vars = ncol(traindatax))

# SHAP of all samples

shaps_rsf_df <- traindatax %>%

pmap_dfr(

function(...) {

current <- tibble(...)

set.seed()

shapi_rsf <- survex::predict_parts(

exper_rsf,

new_observation = current,

N = 100,

calculation_method = "kernelshap"

)

return(shapi_rsf$result %>% rownames_to_column("Time"))

}

)

shaps_rsf_df2 <- shaps_rsf_df %>%

mutate(id = rep(1:nrow(traindatax), each = length(itps))) %>%

select(id, Time, everything()) %>%

pivot_longer(cols = -c(1,2), values_to = "shapley")

shapleyimp_rsf <- shaps_rsf_df2 %>%

group_by(name) %>%

summarise(shapley.abs.mean = mean(abs(shapley), na.rm = T)) %>%

arrange(shapley.abs.mean) %>%

mutate(name = as_factor(name))

flname <- colnames(traindatax)[c(2,4,8)]

lxname <- colnames(traindatax)[-c(2,4,8)]

library(ggh4x)

traindatax %>%

mutate(id = 1:n()) %>%

select(id, all_of(flname)) %>%

pivot_longer(cols = -1) %>%

left_join(shaps_rsf_df2, by = c("id", "name")) %>%

mutate(Time = as_factor(Time)) %>%

ggplot(aes(x = interaction(value, name), y = shapley)) +

geom_boxplot(aes(fill = name), show.legend = F) +

geom_hline(yintercept = 0, color = "grey10") +

scale_x_discrete(NULL, guide = "axis_nested") +

scale_colour_viridis_c() +

labs(x = "") +

facet_wrap(~Time, ncol = 1, scales = "free_y") +

theme_bw() +

theme(axis.text.x = element_text(angle = 30, hjust = 1),

legend.position = "bottom")

traindatax %>%

mutate(id = 1:n()) %>%

select(id, all_of(lxname)) %>%

pivot_longer(cols = -1) %>%

left_join(shaps_rsf_df2, by = c("id", "name")) %>%

mutate(Time = as_factor(Time)) %>%

dplyr::group_by(name) %>%

dplyr::mutate(

value = (value - min(value)) / (max(value) - min(value)),

name = factor(name, levels = levels(shapleyimp_rsf$name))

) %>%

dplyr::arrange(value) %>%

dplyr::ungroup() %>%

ggplot(aes(x = shapley, y = name, color = value)) +

ggbeeswarm::geom_quasirandom(width = 0.2) +

scale_color_gradient(

low = "red",

high = "blue",

breaks = c(0, 1),

labels = c("Low", "High"),

guide = guide_colorbar(barwidth = 0.5,

barheight = length(lxname)*2,

ticks = F,

title.position = "right",

title.hjust = 0.5)

) +

labs(x = "SHAP value", color = "Feature value") +

facet_wrap(~Time) +

theme_bw() +

theme(legend.title = element_text(angle = -90))

**mlr3proba-gbm**

# Load the package

library(tidyverse)

library(survival)

library(mlr3verse)

# remotes::install_github("mlr-org/mlr3proba")

library(mlr3proba)

# remotes::install_github("mlr-org/mlr3extralearners@*release")

library(mlr3extralearners)

source("tidyfuncs4sa.R")

# file.choose()

# Read the data

gbsg <- readr::read_csv("GBSG.csv")

colnames(gbsg)

# Correct the variable types.

# Convert categorical variables to factors

for(i in c(3, 5, 9)){

gbsg[[i]] <- factor(gbsg[[i]])

}

# Exclude variables - irrelevant variables

gbsg$pid <- NULL

# Exclude samples - samples with missing values, samples with non-positive time

# gbsg <- na.omit(gbsg) # Exclude samples with missing values in any variable

gbsg <- gbsg %>%

drop_na(age) %>% # Exclude samples with missing values in specified variables

filter(rfstime > 0) # Exclude samples with non-positive time

# Data overview.

skimr::skim(gbsg)

DataExplorer::plot_correlation(gbsg)

# Time points of interest

sort(unique(gbsg$rfstime))

itps <- c(365 * c(1, 3, 5))

itps

table(cut(gbsg$rfstime, c(0, itps, Inf)))

###################################################

# Data splitting to construct task objects

set.seed(42)

datasplit <- rsample::initial_split(

gbsg, prop = 0.8, strata = rfstime, breaks = 10

)

traindata <- rsample::training(datasplit)

testdata <- rsample::testing(datasplit)

# Comparison of survival curves for the split data

gbsg2 <- gbsg

gbsg2$set <- "test"

gbsg2$set[datasplit$in_id] <- "train"

gbsg2$set <- factor(gbsg2$set)

sfit_set <- survfit(Surv(rfstime, status) ~ set, data=gbsg2)

survminer::ggsurvplot(

sfit_set,

pval=TRUE,

pval.coord = c(0.1, 0.8),

risk.table=TRUE,

ggtheme = theme_minimal()

)

# Data preprocessing

library(recipes)

datarecipe_gbm <- recipe(rfstime + status ~ ., traindata) %>%

step_dummy(all_nominal_predictors()) %>%

prep()

datarecipe_gbm

# Process the training set and test set accordingly

traindata2 <- bake(datarecipe_gbm, new_data = NULL) %>%

dplyr::select(rfstime, status, everything())

colnames(traindata2)

testdata2 <- bake(datarecipe_gbm, new_data = testdata) %>%

dplyr::select(rfstime, status, everything())

colnames(testdata2)

# The training of the task object

task_train <- as_task_surv(

traindata2,

time = "rfstime",

event = "status",

type = "right"

)

task_train

# The test of the task object

task_test <- as_task_surv(

testdata2,

time = "rfstime",

event = "status",

type = "right"

)

task_test

###################################################

# gbm model

# https://mlr3extralearners.mlr-org.com/reference/mlr_learners_surv.gbm.html

# Set up the model

learner_gbm_0 <- lrn(

"surv.gbm",

n.trees = to_tune(100, 150),

interaction.depth = to_tune(1, 2),

n.minobsinnode = to_tune(5, 10),

shrinkage = to_tune(0.001, 0.1)

)

learner_gbm_0

learner_gbm_1 <- ppl(

"distrcompositor",

learner = learner_gbm_0,

estimator = "kaplan",

form = "ph"

)

learner_gbm_1

learner_gbm_2 <- as_learner(

learner_gbm_1

)

learner_gbm_2

# Hyperparameter tuning settings

learner_gbm <- auto_tuner(

tuner = tnr("grid_search", resolution = 3, batch_size = 3),

learner = learner_gbm_2,

resampling = rsmp("cv", folds = 5),

measure = msr("surv.cindex"),

terminator = trm("none")

)

learner_gbm

# Multi-core parallelism

future::plan("multisession")

Training

set.seed(42)

learner_gbm$train(task_train)

# Model overivew

learner_gbm

learner_gbm$tuning_result

learner_gbm$tuning_instance

autoplot(learner_gbm$tuning_instance)

# learner_gbm$tuning_instance$archive

# learner_gbm$tuning_instance$result_learner_param_vals

learner_gbm$learner$model$surv.gbm$model

###################################################

# Predict the training set

predtrain_gbm <- learner_gbm$predict(task_train)

predtrain_gbm

# cindex

predtrain_gbm$score(msrs(c("surv.cindex")))

# Predict the probability of survival at a specified point in time

predprobtrain_gbm <-

predtrain_gbm$distr[

1:nrow(traindata2)

]$survival(itps) %>%

t() %>%

as.data.frame() %>%

mutate(model = "gbm",

dataset = "train",

time = traindata2$rfstime,

status = traindata2$status)

# Evaluation of the model’s performance on the training set

evaltrain_gbm <- eval4sa(

predprob = predprobtrain_gbm,

preddata = traindata2,

etime = "rfstime",

estatus = "status",

model = "gbm",

dataset = "train",

timepoints = itps,

plotcalimethod = "quantile",

bw4nne = NULL,

q4quantile = 5

)

evaltrain_gbm$auc

evaltrain_gbm$roc

evaltrain_gbm$rocplot

evaltrain_gbm$brierscore

evaltrain_gbm$brierscoretest

evaltrain_gbm$calibration

evaltrain_gbm$calibrationplot

# The decision curve at a specified point in time for the training set

tp <- 365*5

tpat <- which(itps == tp)

data.frame(time = traindata2$rfstime,

status = traindata2$status,

Model = 1-predprobtrain_gbm[[tpat]])%>%

dcurves::dca(

Surv(time, status) ~ Model,

data = .,

time = tp,

label = list(Model = "gbm"), # gbm can be replaced with its own model name

thresholds = 0:100 / 100 # The scope can be changed

) %>%

plot() +

labs(title = "DCA on traindata")

# Predict the test set

predtest_gbm <- learner_gbm$predict(task_test)

predtest_gbm

predtest_gbm$score(msrs(c("surv.cindex")))

# Predict the probability of survival at a specified point in time

predprobtest_gbm <-

predtest_gbm$distr[

1:nrow(testdata2)

]$survival(itps) %>%

t() %>%

as.data.frame() %>%

mutate(model = "gbm",

dataset = "test",

time = testdata2$rfstime,

status = testdata2$status)

# Evaluation of the model’s performance on the test setPredict the test set

evaltest_gbm <- eval4sa(

predprob = predprobtest_gbm,

preddata = testdata2,

etime = "rfstime",

estatus = "status",

model = "gbm",

dataset = "test",

timepoints = itps,

plotcalimethod = "quantile",

bw4nne = NULL,

q4quantile = 3

)

evaltest_gbm$auc

evaltest_gbm$roc

evaltest_gbm$rocplot

evaltest_gbm$brierscore

evaltest_gbm$brierscoretest

evaltest_gbm$calibration

evaltest_gbm$calibrationplot

# The decision curve at a specified point in time for a test set set

tp <- 365*5

tpat <- which(itps == tp)

data.frame(time = testdata2$rfstime,

status = testdata2$status,

Model = 1-predprobtest_gbm[[tpat]])%>%

dcurves::dca(

Surv(time, status) ~ Model,

data = .,

time = tp,

label = list(Model = "gbm"), # gbm can be replaced with its own model name

thresholds = 0:100 / 100 # The scope can be changed

) %>%

plot() +

labs(title = "DCA on testdata")

# Save the results for comparison

save(predtrain_gbm,

predprobtrain_gbm,

evaltrain_gbm,

predtest_gbm,

predprobtest_gbm,

evaltest_gbm,

file = ".\\mlsa\\gbm.RData")

# Save the results for use in the SHINY Web Calculator

traindata4gbsg <- traindata

datarecipe_gbm4gbsg <- datarecipe_gbm

learner_gbm4gbsg <- learner_gbm

save(traindata4gbsg,

datarecipe_gbm4gbsg,

learner_gbm4gbsg,

file = ".\\mlsashiny\\gbm.RData")

#############################################

# Model explanation

# The independent variable section

colnames(traindata2)

traindatax <- traindata2[, 3:ncol(traindata2)]

colnames(traindatax)

# Interpreter – Based on the training set, no point in time can be specified

exper_gbm <- survex::explain_survival(

learner_gbm,

data = traindatax,

y = survival::Surv(

time = traindata2$rfstime,

event = traindata2$status

),

predict_function = risk_pred,

predict_survival_function = surv_pred,

predict_cumulative_hazard_function = chf_pred,

label = "gbm",

times = itps

)

# Variable importance

set.seed()

vip_gbm <- survex::model_parts(

exper_gbm,

type = "ratio",

N = 100

)

plot(vip_gbm, max_vars = ncol(traindatax)+1)

# Variable importance bar chart of tick points

vip_gbm$result %>%

filter(`_permutation_` == 0) %>%

rename("Time" = "_times_") %>%

select(Time, all_of(colnames(traindatax))) %>%

pivot_longer(cols = -1) %>%

mutate(name = tidytext::reorder_within(name, value, Time)) %>%

ggplot(aes(x = value, y = name)) +

geom_col(fill = "skyblue") +

geom_vline(xintercept = 1) +

tidytext::scale_y_reordered() +

labs(x = "importance", y = "") +

facet_wrap(~Time, scales = "free_y", labeller = "label_both") +

theme_minimal()

# Partial dependency graphs

set.seed()

partial_gbm <- survex::model_profile(

exper_gbm,

variables = c("pgr", "hormon_X1"),

# Specified as a categorical variable

categorical_variables = c("hormon_X1"),

N = 100,

grid_points = 11

)

plot(partial_gbm, facet_ncol = 1)

# Single-shot prediction decomposition

set.seed()

shap_gbm <- survex::predict_parts(

exper_gbm,

new_observation = traindatax[1, ], # 1st sample

N = 100,

calculation_method = "kernelshap"

)

plot(shap_gbm, max_vars = ncol(traindatax))

# SHAP of all samples

shaps_gbm_df <- traindatax %>%

pmap_dfr(

function(...) {

current <- tibble(...)

set.seed()

shapi_gbm <- survex::predict_parts(

exper_gbm,

new_observation = current,

N = 100,

calculation_method = "kernelshap"

)

return(shapi_gbm$result %>% rownames_to_column("Time"))

}

)

shaps_gbm_df2 <- shaps_gbm_df %>%

mutate(id = rep(1:nrow(traindatax), each = length(itps))) %>%

select(id, Time, everything()) %>%

pivot_longer(cols = -c(1,2), values_to = "shapley")

shapleyimp_gbm <- shaps_gbm_df2 %>%

group_by(name) %>%

summarise(shapley.abs.mean = mean(abs(shapley), na.rm = T)) %>%

arrange(shapley.abs.mean) %>%

mutate(name = as_factor(name))

flname <- colnames(traindatax)[c(2,4,8)]

lxname <- colnames(traindatax)[-c(2,4,8)]

library(ggh4x)

traindatax %>%

mutate(id = 1:n()) %>%

select(id, all_of(flname)) %>%

pivot_longer(cols = -1) %>%

left_join(shaps_gbm_df2, by = c("id", "name")) %>%

mutate(Time = as_factor(Time)) %>%

ggplot(aes(x = interaction(value, name), y = shapley)) +

geom_boxplot(aes(fill = name), show.legend = F) +

geom_hline(yintercept = 0, color = "grey10") +

scale_x_discrete(NULL, guide = "axis_nested") +

scale_colour_viridis_c() +

labs(x = "") +

facet_wrap(~Time, ncol = 1, scales = "free_y") +

theme_bw() +

theme(axis.text.x = element_text(angle = 30, hjust = 1),

legend.position = "bottom")

traindatax %>%

mutate(id = 1:n()) %>%

select(id, all_of(lxname)) %>%

pivot_longer(cols = -1) %>%

left_join(shaps_gbm_df2, by = c("id", "name")) %>%

mutate(Time = as_factor(Time)) %>%

dplyr::group_by(name) %>%

dplyr::mutate(

value = (value - min(value)) / (max(value) - min(value)),

name = factor(name, levels = levels(shapleyimp_gbm$name))

) %>%

dplyr::arrange(value) %>%

dplyr::ungroup() %>%

ggplot(aes(x = shapley, y = name, color = value)) +

ggbeeswarm::geom_quasirandom(width = 0.2) +

scale_color_gradient(

low = "red",

high = "blue",

breaks = c(0, 1),

labels = c("Low", "High"),

guide = guide_colorbar(barwidth = 0.5,

barheight = length(lxname)*2,

ticks = F,

title.position = "right",

title.hjust = 0.5)

) +

labs(x = "SHAP value", color = "Feature value") +

facet_wrap(~Time) +

theme_bw() +

theme(legend.title = element_text(angle = -90))

**mlr3proba-xgboost**

# Load the package

setwd("C:/Construction of machine learning prognostic models")

library("tidyverse")

library("rlang")

library("devtools")

library("tidymodels")

library("usethis")

library("mlr3proba")

library("survival")

library("xgboost")

library("mlr3verse")

library("mlr3extralearners")

library("skimr")

library("survival")

library("DataExplorer")

library("survminer")

library("GGally")

library("ggplot2")

library("dcurves")

library("ggbeeswarm")

library("ggh4x")

source("tidyfuncs4sa.R")

# Read the data

gbsg <- readr::read_csv("data1.csv")

gbsg <- gbsg[1:9]

colnames(gbsg)

# Correct the variable types.

# Convert categorical variables to factors

for(i in c(1:7)){

gbsg[[i]] <- factor(gbsg[[i]])

}

# Exclude variables - irrelevant variables

gbsg$pid <- NULL

# Exclude samples - samples with missing values, samples with non-positive time

# gbsg <- na.omit(gbsg) # Exclude samples with missing values in any variable

gbsg <- gbsg %>%

drop_na(Age) %>% # Exclude samples with missing values in specified variables

filter(rfstime > 0) # Exclude samples with non-positive time

# Data overview.

skimr::skim(gbsg)

DataExplorer::plot_correlation(gbsg)

# Time points of interest

sort(unique(gbsg$rfstime))

itps <- c(365 * c(1, 3, 5))

itps

table(cut(gbsg$rfstime, c(0, itps, Inf)))

###################################################

# Data splitting to construct task objects

set.seed(42)

datasplit <- rsample::initial_split(

gbsg, prop = 0.8, strata = rfstime, breaks = 10

)

traindata <- rsample::training(datasplit)

testdata <- rsample::testing(datasplit)

# Comparison of survival curves for the split data

gbsg2 <- gbsg

gbsg2$set <- "test"

gbsg2$set[datasplit$in_id] <- "train"

gbsg2$set <- factor(gbsg2$set)

sfit_set <- survfit(Surv(rfstime, status) ~ set, data=gbsg2)

survminer::ggsurvplot(

sfit_set,

pval=TRUE,

pval.coord = c(0.1, 0.8),

risk.table=TRUE,

ggtheme = theme_minimal()

)

# Data preprocessing

library(recipes)

datarecipe_xgboost <- recipe(rfstime + status ~ ., traindata) %>%

step_dummy(all_nominal_predictors()) %>%

prep()

datarecipe_xgboost

# Process the training set and test set accordingly

traindata2 <- bake(datarecipe_xgboost, new_data = NULL) %>%

dplyr::select(rfstime, status, everything())

colnames(traindata2)

testdata2 <- bake(datarecipe_xgboost, new_data = testdata) %>%

dplyr::select(rfstime, status, everything())

colnames(testdata2)

task_train <- as_task_surv(

traindata2,

time = "rfstime",

event = "status",

type = "right"

)

task_train

# The test of the task object

task_test <- as_task_surv(

testdata2,

time = "rfstime",

event = "status",

type = "right"

)

task_test

###################################################

# xgboost model

# https://mlr3extralearners.mlr-org.com/reference/mlr_learners_surv.xgboost.html

# Set up the model

learner_xgboost_0 <- lrn(

"surv.xgboost.cox",

nrounds = to_tune(100, 500),

max_depth = to_tune(1, 5),

eta = to_tune(1e-4, 1)

)

learner_xgboost_0

learner_xgboost_1 <- ppl(

"distrcompositor",

learner = learner_xgboost_0,

estimator = "kaplan",

form = "ph"

)

learner_xgboost_1

learner_xgboost_2 <- as_learner(

learner_xgboost_1

)

learner_xgboost_2

# Hyperparameter tuning settings

learner_xgboost <- auto_tuner(

tuner = tnr("random_search", batch_size = 4),

resampling = rsmp("cv", folds = 5),

measure = msr("surv.cindex"),

terminator = trm("evals", n_evals = 40),

learner = learner_xgboost_0)

learner_xgboost

learner_xgboost$train(task_train)

# Multi-core parallelism

future::plan("multisession")

Training

set.seed(42)

learner_xgboost$train(task_train)

# Model overivew

learner_xgboost

learner_xgboost$tuning_result

learner_xgboost$tuning_instance

autoplot(learner_xgboost$tuning_instance)

# learner_xgboost$tuning_instance$archive

# learner_xgboost$tuning_instance$result_learner_param_vals

learner_xgboost$learner$model$surv.xgboost$model

###################################################

# Predict the training set

predtrain_xgboost <- learner_xgboost$predict(task_train)

predtrain_xgboost

# cindex

predtrain_xgboost$score(msrs(c("surv.cindex")))

# Predict the probability of survival at a specified point in time

predprobtrain_xgboost <-

predtrain_xgboost$distr[

1:nrow(traindata2)

]$survival(itps) %>%

t() %>%

as.data.frame() %>%

mutate(model = "xgboost",

dataset = "train",

time = traindata2$rfstime,

status = traindata2$status)

# Evaluation of the model’s performance on the training set

evaltrain_xgboost <- eval4sa(

predprob = predprobtrain_xgboost,

preddata = traindata2,

etime = "rfstime",

estatus = "status",

model = "xgboost",

dataset = "train",

timepoints = itps,

plotcalimethod = "quantile",

bw4nne = NULL,

q4quantile = 5

)

evaltrain_xgboost$auc

evaltrain_xgboost$roc

evaltrain_xgboost$rocplot

evaltrain_xgboost$brierscore

evaltrain_xgboost$brierscoretest

evaltrain_xgboost$calibration

evaltrain_xgboost$calibrationplot

# The decision curve at a specified point in time for the training set

tp <- 365*5

tpat <- which(itps == tp)

data.frame(time = traindata2$rfstime,

status = traindata2$status,

Model = 1-predprobtrain_xgboost[[tpat]])%>%

dcurves::dca(

Surv(time, status) ~ Model,

data = .,

time = tp,

label = list(Model = "xgboost"), # xgboost can be replaced with its own model name

thresholds = 0:100 / 100 # The scope can be changed

) %>%

plot() +

labs(title = "DCA on traindata")

# Predict the test set

predtest_xgboost <- learner_xgboost$predict(task_test)

predtest_xgboost

predtest_xgboost$score(msrs(c("surv.cindex")))

# Predict the probability of survival at a specified point in time

predprobtest_xgboost <-

predtest_xgboost$distr[

1:nrow(testdata2)

]$survival(itps) %>%

t() %>%

as.data.frame() %>%

mutate(model = "xgboost",

dataset = "test",

time = testdata2$rfstime,

status = testdata2$status)

# Evaluation of the model’s performance on the test setPredict the test set

evaltest_xgboost <- eval4sa(

predprob = predprobtest_xgboost,

preddata = testdata2,

etime = "rfstime",

estatus = "status",

model = "xgboost",

dataset = "test",

timepoints = itps,

plotcalimethod = "quantile",

bw4nne = NULL,

q4quantile = 3

)

evaltest_xgboost$auc

evaltest_xgboost$roc

evaltest_xgboost$rocplot

evaltest_xgboost$brierscore

evaltest_xgboost$brierscoretest

evaltest_xgboost$calibration

evaltest_xgboost$calibrationplot

# The decision curve at a specified point in time for a test set set

tp <- 365*5

tpat <- which(itps == tp)

data.frame(time = testdata2$rfstime,

status = testdata2$status,

Model = 1-predprobtest_xgboost[[tpat]])%>%

dcurves::dca(

Surv(time, status) ~ Model,

data = .,

time = tp,

label = list(Model = "xgboost"), # xgboost can be replaced with its own model name

thresholds = 0:100 / 100 # The scope can be changed

) %>%

plot() +

labs(title = "DCA on testdata")

# Save the results for comparison

save(predtrain_xgboost,

predprobtrain_xgboost,

evaltrain_xgboost,

predtest_xgboost,

predprobtest_xgboost,

evaltest_xgboost,

file = ".\\mlsa\\xgboost.RData")

# Save the results for use in the SHINY Web Calculator

traindata4gbsg <- traindata

datarecipe_xgboost4gbsg <- datarecipe_xgboost

learner_xgboost4gbsg <- learner_xgboost

save(traindata4gbsg,

datarecipe_xgboost4gbsg,

learner_xgboost4gbsg,

file = "C:/Construction of machine learning prognostic models/mlsa/xgboost.RData")

#############################################

# Model explanation

# The independent variable section

colnames(traindata2)

traindatax <- traindata2[, 3:ncol(traindata2)]

colnames(traindatax)

# Interpreter – Based on the training set, no point in time can be specified

exper_xgboost <- survex::explain_survival(

learner_xgboost,

data = traindatax,

y = survival::Surv(

time = traindata2$rfstime,

event = traindata2$status

),

predict_function = risk_pred,

predict_survival_function = surv_pred,

predict_cumulative_hazard_function = chf_pred,

label = "xgboost",

times = itps

)

# Variable importance

set.seed(42)

vip_xgboost <- survex::model_parts(

exper_xgboost,

type = "ratio",

N = 100

)

plot(vip_xgboost, max_vars = ncol(traindatax)+1)

# Variable importance bar chart of tick points

vip_xgboost$result %>%

filter(`_permutation_` == 0) %>%

rename("Time" = "_times_") %>%

select(Time, all_of(colnames(traindatax))) %>%

pivot_longer(cols = -1) %>%

mutate(name = tidytext::reorder_within(name, value, Time)) %>%

ggplot(aes(x = value, y = name)) +

geom_col(fill = "skyblue") +

geom_vline(xintercept = 1) +

tidytext::scale_y_reordered() +

labs(x = "importance", y = "") +

facet_wrap(~Time, scales = "free_y", labeller = "label_both") +

theme_minimal()

# Partial dependency graphs

set.seed(42)

partial_xgboost <- survex::model_profile(

exper_xgboost,

variables = c("pgr", "hormon_X1"),

# Specified as a categorical variable

categorical_variables = c("hormon_X1"),

N = 100,

grid_points = 11

)

plot(partial_xgboost, facet_ncol = 1)

# Single-shot prediction decomposition

set.seed(42)

shap_xgboost <- survex::predict_parts(

exper_xgboost,

new_observation = traindatax[1, ], # 1st sample

N = 100,

calculation_method = "kernelshap"

)

plot(shap_xgboost, max_vars = ncol(traindatax))

# SHAP of all samples

shaps_xgboost_df <- traindatax %>%

pmap_dfr(

function(...) {

current <- tibble(...)

set.seed(42)

shapi_xgboost <- survex::predict_parts(

exper_xgboost,

new_observation = current,

N = 100,

calculation_method = "kernelshap"

)

return(shapi_xgboost$result %>% rownames_to_column("Time"))

}

)

shaps_xgboost_df2 <- shaps_xgboost_df %>%

mutate(id = rep(1:nrow(traindatax), each = length(itps))) %>%

select(id, Time, everything()) %>%

pivot_longer(cols = -c(1,2), values_to = "shapley")

shapleyimp_xgboost <- shaps_xgboost_df2 %>%

group_by(name) %>%

summarise(shapley.abs.mean = mean(abs(shapley), na.rm = T)) %>%

arrange(shapley.abs.mean) %>%

mutate(name = as_factor(name))

flname <- colnames(traindatax)[c(2,4,8)]

lxname <- colnames(traindatax)[-c(2,4,8)]

library(ggh4x)

traindatax %>%

mutate(id = 1:n()) %>%

select(id, all_of(flname)) %>%

pivot_longer(cols = -1) %>%

left_join(shaps_xgboost_df2, by = c("id", "name")) %>%

mutate(Time = as_factor(Time)) %>%

ggplot(aes(x = interaction(value, name), y = shapley)) +

geom_boxplot(aes(fill = name), show.legend = F) +

geom_hline(yintercept = 0, color = "grey10") +

scale_x_discrete(NULL, guide = "axis_nested") +

scale_colour_viridis_c() +

labs(x = "") +

facet_wrap(~Time, ncol = 1, scales = "free_y") +

theme_bw() +

theme(axis.text.x = element_text(angle = 30, hjust = 1),

legend.position = "bottom")

traindatax %>%

mutate(id = 1:n()) %>%

select(id, all_of(lxname)) %>%

pivot_longer(cols = -1) %>%

left_join(shaps_xgboost_df2, by = c("id", "name")) %>%

mutate(Time = as_factor(Time)) %>%

dplyr::group_by(name) %>%

dplyr::mutate(

value = (value - min(value)) / (max(value) - min(value)),

name = factor(name, levels = levels(shapleyimp_xgboost$name))

) %>%

dplyr::arrange(value) %>%

dplyr::ungroup() %>%

ggplot(aes(x = shapley, y = name, color = value)) +

ggbeeswarm::geom_quasirandom(width = 0.2) +

scale_color_gradient(

low = "red",

high = "blue",

breaks = c(0, 1),

labels = c("Low", "High"),

guide = guide_colorbar(barwidth = 0.5,

barheight = length(lxname)*2,

ticks = F,

title.position = "right",

title.hjust = 0.5)

) +

labs(x = "SHAP value", color = "Feature value") +

facet_wrap(~Time) +

theme_bw() +

theme(legend.title = element_text(angle = -90))

**R package**

# Evaluation functions

eval4sa <- function(

predprob, preddata, etime, estatus, model, dataset, timepoints,

plotcalimethod, bw4nne, q4quantile

) {

# predprob = predprobtrain_coxph

# preddata = traindata2

# etime = "rfstime"

# estatus = "status"

# model = "coxph"

# dataset = "train"

# timepoints = interesttimepoints

# plotcalimethod = "quantile"

# bw4nne = NULL

# q4quantile = 5

# Survival object

survobj <- as.formula(

paste0('Surv(', etime, ', ', estatus, ') ~ 1')

)

# container

aucdf <- list()

rocdf <- list()

auc2df <- list()

roc2df <- list()

bsdf <- list()

bscdf <- list()

caldf <- list()

# Calculated on a point-in-time basis

for (i in seq_along(timepoints)) {

# i=1

datai <- data.frame(vi = 1-predprob[,i])

colnames(datai) <- model

################################################

set.seed(511543)

score_obj <- riskRegression::Score(

as.list(datai),

formula = survobj,

data = preddata,

metrics=c("auc", "brier"),

summary = c("IPA"),

plots = c("roc", "calibrate"),

times = timepoints[i],

conf.int = T

)

aucdf[[i]] <- score_obj$AUC$score %>%

mutate(dataset = dataset)

rocdf[[i]] <- score_obj$ROC$plotframe %>%

mutate(dataset = dataset)

bsdf[[i]] <- score_obj$Brier$score %>%

mutate(dataset = dataset)

bscdf[[i]] <- score_obj$Brier$contrasts %>%

mutate(dataset = dataset)

plotcali <- riskRegression::plotCalibration(

score_obj,

cens.method = "local",

method = plotcalimethod,

bandwidth = bw4nne,

q = q4quantile,

plot = F

)

caldf[[i]] <- plotcali$plotFrames[[1]] %>%

mutate(dataset = dataset,

times = timepoints[i],

model = model)

}

#################################################

rocdf_plus1 <- data.frame(

model = model,

times = timepoints,

risk = -Inf,

TPR = 0,

FPR = 0,

dataset = dataset

)

# rocdf_plus2 <- data.frame(

# model = model,

# times = timepoints,

# risk = -Inf,

# TPR = 1,

# FPR = 1,

# dataset = dataset

# )

dataauc <- bind_rows(aucdf)

# dataroc <- bind_rows(rocdf, rocdf_plus1, rocdf_plus2) %>%

dataroc <- bind_rows(rocdf, rocdf_plus1) %>%

arrange(TPR)

plotroc <- dataroc %>%

left_join(dataauc, by = c("model", "times", "dataset")) %>%

mutate(tauc = paste0("T=", times, ", AUC=", round(AUC, 3),

"(", round(lower, 3), "~",

round(upper, 3), ")"),

tauc = forcats::as_factor(tauc)) %>%

ggplot(aes(x = FPR, y = TPR, group = tauc, color = tauc)) +

geom_line(linewidth = 1) +

geom_abline(color = "grey") +

scale_x_continuous(expand = c(0, 0)) +

scale_y_continuous(expand = c(0, 0)) +

labs(x = "1-Specificity", y = "Sensitivity", color = "") +

theme_bw() +

theme(legend.position = c(1,0),

legend.justification = c(1,0),

legend.background = element_blank(),

legend.key = element_blank())

#################################################

databs <- bind_rows(bsdf)

databsc <- bind_rows(bscdf)

datacal <- bind_rows(caldf)

rownames(datacal) <- NULL

plotcal <- datacal %>%

mutate(t = paste0("T=", times),

t = forcats::as_factor(t)) %>%

ggplot(aes(x = Pred, y = Obs, group = t, color = t)) +

geom_line(linewidth = 1) +

geom_point(size = 3,

alpha = ifelse(plotcalimethod == "nne", 0, 1)) +

geom_abline() +

scale_x_continuous(limits = c(0, 1), expand = c(0, 0)) +

scale_y_continuous(limits = c(0, 1), expand = c(0, 0)) +

labs(x = "Predicted risk",

y = "Estimated actual risk",

color = "") +

theme_bw() +

theme(legend.position = c(1,0),

legend.justification = c(1,0),

legend.background = element_blank(),

legend.key = element_blank())

return(list(

auc = dataauc,

roc = dataroc,

rocplot = plotroc,

brierscore = databs,

brierscoretest = databsc,

calibration = datacal,

calibrationplot = plotcal

))

}

##############################################################

##############################################################

##############################################################

# Interpreter functions

risk_pred <- function(model, newdata){

model$predict_newdata(newdata)$crank

}

surv_pred <- function(model, newdata, times){

t(model$predict_newdata(newdata)$distr$survival(times))

}

chf_pred <- function(model, newdata, times){

t(model$predict_newdata(newdata)$distr$cumHazard(times))

}

**Model comparison**

# Load the package

library(tidyverse)

# Load the evaluation results for each model

evalfiles <- list.files(".\\mlsa\\", full.names = T)

lapply(evalfiles, load, .GlobalEnv)

# The number of models that are compared side-by-side

nmodels <- 6

cols4model <- rainbow(nmodels) # Unify the color scheme of the model

#############################################################

# The results of the evaluation of each model on the test set

names(evaltest_svm)

# The AUC of each model at each point in time of the test set

testauc_all <- bind_rows(

lapply(list(

evaltest_gbm, evaltest_rpart,

evaltest_rsf, evaltest_lasso,

evaltest_svm, evaltest_xgboost),

"[[",

"auc")

)#valtest_nn,

testauc_all

testauc_min <- testauc_all %>%

group_by(times) %>%

slice_min(AUC, n = 1)

testauc_max <- testauc_all %>%

group_by(times) %>%

slice_max(AUC, n = 1)

testauc_all %>%

ggplot(aes(x = times, y = AUC, color = model)) +

geom_point() +

geom_line(aes(group = model)) +

# ggrepel::geom_text_repel(testauc_max,

# mapping = aes(label = model),

# nudge_y = 0.05,

# show.legend = F) +

# ggrepel::geom_text_repel(testauc_min,

# mapping = aes(label = model),

# nudge_y = -0.05,

# show.legend = F) +

scale_x_continuous(breaks = unique(testauc_all$times)) +

scale_color_manual(values = c(cols4model)) +

labs(x = "Time") +

theme_bw()

#############################

# The BS of each model pair at each point in time of the test set

testbs_all <- bind_rows(

lapply(list(evaltest_gbm, evaltest_rpart,

evaltest_rsf,evaltest_lasso,

evaltest_svm, evaltest_xgboost),

"[[",

"brierscore")

)

testbs_all

testbs_min <- testbs_all %>%

filter(model != "Null model") %>%

group_by(times) %>%

slice_min(Brier, n = 1)

testbs_max <- testbs_all %>%

filter(model != "Null model") %>%

group_by(times) %>%

slice_max(Brier, n = 1)

testbs_all %>%

filter(model != "Null model") %>%

ggplot(aes(x = times, y = Brier, color = model)) +

geom_point() +

geom_line(aes(group = model)) +

scale_x_continuous(breaks = unique(testbs_all$times)) +

scale_color_manual(values = c(cols4model)) +

labs(x = "Time") +

theme_bw()

#############################################################

# ROC of each model on the test set

rocsall <- bind_rows(

lapply(list(evaltest_gbm, evaltest_rpart,

evaltest_rsf,evaltest_lasso,

evaltest_svm, evaltest_xgboost),

"[[",

"roc")

)

rocsall %>%

left_join(testauc_all, by = c("model", "times")) %>%

filter(times == 365) %>% # The time point can be replaced) %>% # The time point can be replaced

mutate(mtAUC = paste0(model, ", T=", times,

", AUC=", round(AUC, 4))) %>%

ggplot(aes(x = FPR,

y = TPR,

color = forcats::as_factor(mtAUC))) +

geom_path(linewidth = 1.2) +

geom_abline(slope = 1, intercept = 0) +

scale_x_continuous(expand = c(0,0)) +

scale_y_continuous(expand = c(0,0)) +

scale_color_manual(values = c(cols4model)) +

labs(color = "", x = "1 - Specificity", y = "Sensitivity") +

theme_bw() +

theme(legend.position = c(1,0),

legend.justification = c(1,0),

legend.background = element_blank(),

legend.key = element_blank())

rocsall %>%

left_join(testauc_all, by = c("model", "times")) %>%

filter(times == 1095) %>% # The time point can be replaced) %>% # The time point can be replaced

mutate(mtAUC = paste0(model, ", T=", times,

", AUC=", round(AUC, 4))) %>%

ggplot(aes(x = FPR,

y = TPR,

color = forcats::as_factor(mtAUC))) +

geom_path(linewidth = 1.2) +

geom_abline(slope = 1, intercept = 0) +

scale_x_continuous(expand = c(0,0)) +

scale_y_continuous(expand = c(0,0)) +

scale_color_manual(values = c(cols4model)) +

labs(color = "", x = "1 - Specificity", y = "Sensitivity") +

theme_bw() +

theme(legend.position = c(1,0),

legend.justification = c(1,0),

legend.background = element_blank(),

legend.key = element_blank())

rocsall %>%

left_join(testauc_all, by = c("model", "times")) %>%

filter(times == 1825) %>% # The time point can be replaced) %>% # The time point can be replaced

mutate(mtAUC = paste0(model, ", T=", times,

", AUC=", round(AUC, 4))) %>%

ggplot(aes(x = FPR,

y = TPR,

color = forcats::as_factor(mtAUC))) +

geom_path(linewidth = 1.2) +

geom_abline(slope = 1, intercept = 0) +

scale_x_continuous(expand = c(0,0)) +

scale_y_continuous(expand = c(0,0)) +

scale_color_manual(values = c(cols4model)) +

labs(color = "", x = "1 - Specificity", y = "Sensitivity") +

theme_bw() +

theme(legend.position = c(1,0),

legend.justification = c(1,0),

legend.background = element_blank(),

legend.key = element_blank())

#############################################################

# DCA of each model on the test set

predprobtest_all <- bind_rows(

predprobtest_gbm,

predprobtest_rpart,

predprobtest_rsf,

predprobtest_svm,

predprobtest_lasso,

predprobtest_xgboost

)

predprobtest_all

predprobtest_all %>%

select(`365`, time, status, model) %>% # Timepoint 1825 can be replaced

mutate(id = rep(1:nrow(predprobtest_gbm), nmodels),

`365` = 1 - `365`) %>%

pivot_wider(names_from = model, values_from = `365`) %>%

select(-id) %>%

dcurves::dca(

survival::Surv(time, status) ~ .,

data = .,

time = 365, # 60Corresponds to the above

thresholds = 0:100 / 100 # The scope can be changed

) %>%

plot(smooth = T, span = 0.5) +

scale_color_manual(values = c("black", "grey", cols4model)) +

labs(title = "DCA on testdata")

predprobtest_all %>%

select(`1095`, time, status, model) %>% # Timepoint 1825 can be replaced

mutate(id = rep(1:nrow(predprobtest_gbm), nmodels),

`1095` = 1 - `1095`) %>%

pivot_wider(names_from = model, values_from = `1095`) %>%

select(-id) %>%

dcurves::dca(

survival::Surv(time, status) ~ .,

data = .,

time = 1095, # 60Corresponds to the above

thresholds = 0:100 / 100 # The scope can be changed

) %>%

plot(smooth = T, span = 0.5) +

scale_color_manual(values = c("black", "grey", cols4model)) +

labs(title = "DCA on testdata")

predprobtest_all %>%

select(`1825`, time, status, model) %>% # Timepoint 1825 can be replaced

mutate(id = rep(1:nrow(predprobtest_gbm), nmodels),

`1825` = 1 - `1825`) %>%

pivot_wider(names_from = model, values_from = `1825`) %>%

select(-id) %>%

dcurves::dca(

survival::Surv(time, status) ~ .,

data = .,

time = 1825, # 60Corresponds to the above

thresholds = 0:100 / 100 # The scope can be changed

) %>%

plot(smooth = T, span = 0.5) +

scale_color_manual(values = c("black", "grey", cols4model)) +

labs(title = "DCA on testdata")
